# Herbal Amara extract induces gastric fundus relaxation via inhibition of the M2 muscarinic receptor

**DOI:** 10.1111/nmo.14924

**Published:** 2024-09-30

**Authors:** Maria‐Riera Piqué‐Borràs, Johann Röhrl, Gerald Künstle

**Affiliations:** ^1^ Preclinical Research and Development Weleda AG Arlesheim Switzerland

**Keywords:** dyspepsia, gastric fundus, herbal medicine, Phytotherapy, plant extract, signs and symptoms, digestive, stomach diseases

## Abstract

**Background:**

Impaired gastric accommodation is one of the most frequent symptoms of functional dyspepsia. The safety and efficacy of conventional treatments remain to be proven and alternative herbal therapies have been proposed to alleviate gastrointestinal symptoms. This preclinical study examined the role of herbal Amara extract (containing *Artemisia absinthium*, *Centaurium erythraea*, *Cichorium intybus*, *Gentiana lutea*, *Juniperus communis*, *Achillea millefolium*, *Peucedanum ostruthium*, *Salvia officinalis*, and *Taraxacum* extracts) on gastric (fundus) accommodation and the possible implication of muscarinic receptors in its regulation.

**Methods:**

The effect of Amara extract on fundus motility was investigated in organ baths of smooth muscle strips isolated from the fundus of guinea pigs, and the role of the muscarinic receptor pathway was evaluated using functional and radioligand binding assays in cell lines expressing the M2 or M3 muscarinic receptor.

**Key Results:**

Amara extract inhibited carbachol‐induced contraction of guinea pig smooth muscle strips in a dose‐dependent manner. This relaxant effect was not affected by the M3 antagonist J‐104129. Amara extract also inhibited M2, but not M3, receptor activity in CHO‐K1 cells (IC_50_ 219 μg mL^−1^), and specifically bound the M2 receptor (IC_50_ 294 μg mL^−1^). Of the nine herbal components of Amara extract, *Juniperus communis*, *P. ostruthium*, and *Salvia officinalis* inhibited M2 receptor activity (IC_50_ 32.0, 20.8, and 20.1 μg mL^−1^, respectively), and *P. ostruthium* was sufficient to reverse carbachol‐induced ex vivo contraction of guinea pig fundic smooth muscles.

**Conclusion and Inferences:**

Amara extract relaxes gastric smooth muscles by inhibiting the M2 muscarinic receptor. This study suggests the potential benefit of Amara extract for patients with impaired gastric accommodation.

## Graphical abstract

This preclinical study demonstrates the relaxant effect of Amara herbal extract (containing nine different plant extracts) on fundus smooth muscles dissected from guinea pigs. This relaxant effect occurs via inhibition of M2 muscarinic receptor activity. This study reveals the potential benefit of Amara extract for patients with impaired gastric accommodation.

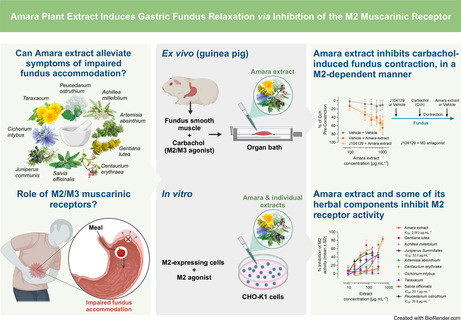

**Key points**

- Herbal Amara extract (containing *Artemisia absinthium*, *Centaurium erythraea*, *Cichorium intybus*, *Gentiana lutea*, *Juniperus communis*, *Achillea millefolium*, *Peucedanum ostruthium*, *Salvia officinalis*, and *Taraxacum* extracts) relaxes gastric smooth muscles, in a model of guinea pig fundus smooth muscle organ bath.
- Amara extract’s relaxant effect involves inhibition of the M2 muscarinic receptor.
- This preclinical study demonstrates the effect of Amara extract on gastric motility in an animal model, and suggests its potential benefit for patients with impaired gastric accommodation.

## 1 INTRODUCTION

Amara drops (Weleda AG, Schwäbisch Gmünd, Germany) is an ethanolic herbal solution composed of nine plant extracts (*Artemisia absinthium*, *Centaurium erythraea*, *Cichorium intybus*, *Gentiana lutea*, *Juniperus communis*, *Achillea millefolium*, *Peucedanum ostruthium*, *Salvia officinalis*, and *Taraxacum*). It is indicated for the treatment of imbalanced production and secretion of digestive juices, and of gastric and intestinal motility disorders and their associated symptoms.^7^ The nine plants composing Amara Drops belong to four groups of bitter traditional medicinal plants known for relieving symptoms of various digestive disorders: (i) Amara pura (*Centaurium erythraea*, *Cichorium intybus*, *Salvia officinalis*, *Taraxacum*), (ii) Amara astringentia (*Juniperus communis*, *Salvia officinalis*), (iii) Amara aromatic (*Artemisia absinthium*, *Achillea millefolium*), and (iv) Amara acria (*P. ostruthium*).^8^, ^9^, ^10^, ^11^, ^12^, ^13^, ^14^, ^15^, ^16^

Gastric and intestinal motility disorders and their associated symptoms, including heartburn, nausea, loss of appetite, and early satiety, are hallmarks of functional dyspepsia. Functional dyspepsia is a debilitating condition affecting 11%–29% of the global population.^17^ It includes a constellation of upper gastrointestinal symptoms, such as bloating, belching, early satiety, postprandial fullness, epigastric pain, epigastric burning, nausea, and vomiting.^18^, ^19^ The etiology of functional dyspepsia is not well defined, and the term functional dyspepsia usually describes dyspepsia symptoms with no identified organic etiology.^18^ Risk factors for functional dyspepsia include female sex, age, *Helicobacter pylori* infection, smoking, genetic factors, and nonsteroidal anti‐inflammatory medication usage.^18^, ^20^ Functional dyspepsia is also associated with (i) psychological distress, particularly anxiety, in a proposed bidirectional gut‐brain axis mechanism, (ii) gastric neuromuscular dysfunction, including delayed gastric emptying and impaired gastric fundus relaxation, (iii) duodenal impairments (inflammation, dysmotility, acid exposure), and (iv) pathogen‐induced gastroenteritis.^18^, ^19^, ^20^

Pharmacological evidence suggests an implication of multiple pathways in functional dyspepsia, including serotonin receptors of the submucosal sensory neurons, muscarinic receptors of gastric smooth muscle cells, and opioid receptors of the vagal afferent pathway.^19^, ^21^, ^22^, ^23^ Hence, the prokinetic agent acotiamide, which acts as an antagonist of M1 and M2 muscarinic receptors, appears to be effective against impaired gastric accommodation.^21^, ^24^ Impaired gastric (fundus) accommodation is one of the most common symptoms of functional dyspepsia. It is described in 40% of patients,^19^ and thus represents a relevant target for intervention. Multiple prokinetic agents and neuromodulators targeting not only the muscarinic but also the serotonin and/or opioid pathways have been indicated for the treatment of functional dyspepsia. However, due to the heterogeneity of disease symptoms, their efficacy remains overall unclear. Besides, several of these treatments were withdrawn or are not recommended in routine due to serious side effects.^18^, ^19^, ^20^, ^23^

Alternative medicine therapies, such as phytotherapy have been proposed and used by approximately 50% of functional dyspepsia patients to alleviate their gastrointestinal symptoms.^18^, ^25^ These herbal remedies include, for example, STW5 (Iberogast, Bayer) and Rikkunshito (TJ‐43, Tsumura and Co).^18^, ^20^, ^23^, ^26^, ^27^, ^28^, ^29^, ^30^, ^31^

Like the Amara Drops, STW5 is a multi‐herbal extract with multiple therapeutic targets relevant to the gastrointestinal symptoms of functional dyspepsia.^22^, ^32^ Placebo‐controlled studies demonstrated the clinical efficacy and safety of STW5 for the treatment of patients with functional dyspepsia,^29^, ^30^, ^32^ while in vitro experiments highlighted its effect on both gastric and intestinal motility.^26^, ^27^, ^28^, ^31^, ^32^, ^33^ Whereas the in vitro effect of STW5 on gastrointestinal pathways is well documented, that of Amara extract remains to be evaluated.

This preclinical study examined the role of Amara extract on fundus accommodation and investigated the possible implication of muscarinic receptors in that process. The effect of Amara extract on fundus motility and relaxation was investigated on smooth muscle strips isolated from the fundus of guinea pigs, and the characterization of the molecular pathway involved was conducted using cellular assays.

This experimental study showed that Amara extract induces fundus relaxation and reduces carbachol‐induced contraction of smooth muscle strips from the fundus of guinea pigs and that this effect likely involves the M2 but not the M3 muscarinic receptors.

## 2 MATERIALS AND METHODS

### 2.1 Herbal extracts

Amara Drops (originally described in the monograph of Commission C entitled “Cichorium/Taraxacum comp.” published in the German Federal Gazette Nr. 99a in June 1986 and amended in the Federal Gazette Nr. 85 in May 1991)^34^ is a 33% (vol) ethanol solution containing the following amounts of tinctures (in g) for 10 g (10.4 mL) of product: 0.15 g ethanolic extract from *Artemisia absinthium*, Herba rec. (1:2.3); 0.075 g ethanolic extract from *Centaurium erythraea*, Herba rec. (1:2.3); 0.6 g ethanolic extract from *Cichorium intybus*, Planta tota rec. (1:2.3); 0.36 g ethanolic decoction from *Gentiana lutea*, Rhizoma and Radix Ø (Ph. Eur. 10/2544, V. 1.2.10); 0.05 g ethanolic infusion from *Juniperus communis*, Summitates Ø (Ph. Eur. 10/2545, V. 1.2.13); 2.0 g ethanolic infusion from *Achillea millefolium* Ø (Ph. Eur. 10/2545, V. 1.2.13); 0.15 g ethanolic decoction from *P. ostruthium*, Rhizoma rec. (1:2.15) (Ph. Eur. 10/2544, V. 1.2.10); 1.0 g ethanolic infusion from Salvia officinalis Ø (Ph. Eur. 10/2545, V. 1.2.13); 0.32 g *Taraxacum*, Planta tota rec. Ø (Ph. Eur. 10/2535, V. 1.1.3).^7^ A recommended dosage of Amara Drops (15 drops or 348 μL) contains a total of 3.97 mg dry herbal extract, including the following amounts of the respective dry herbal components: 152 μg *Artemisia absinthium*, 66 μg *Centaurium erythraea*, 344 μg *Cichorium intybus*, 571 μg *Gentiana lutea*, 37 μg *Juniperus communis*, 1416 μg *Achillea millefolium*, 126 μg *P. ostruthium*, 910 μg Salvia officinalis, and 345 μg *Taraxacum*.

The nine individual extract tinctures were prepared as follows: (1) *Artemisia absinthium*, Herba rec., ethanolic extract 1:2.3 EtOH 30% (m/m); (2) *Centaurium erythraea*, Herba rec., ethanolic extract 1:2.3 EtOH 30% (m/m); (3) *Cichorium intybus*, Planta tota rec., ethanolic extract 1:2.3 EtOH 30% (m/m); (4) *Gentiana lutea*, Rhizoma and Radix, ethanol.Decoctum Ø (Ph. Eur. 10/2544, V. 1.2.10); (5) *Juniperus communis*, Summitates, ethanol.Infusum Ø 1:10 (Ph. Eur. 10/2545, V. 1.2.13) EtOH 30% (m/m); (6) *Achillea millefolium*, ethanol.Infusum Ø 1:10 (Ph. Eur. 10/2545, V. 1.2.13) EtOH 30% (m/m); (7) *P. ostruthium*, Rhizoma rec., ethanol.Decoctum (Ph. Eur. 10/2544, V. 1.2.10) 1:2.15 EtOH 43% (m/m); (8) *Salvia officinalis*, ethanol.Infusum Ø 1:10 (Ph. Eur. 10/2545, V. 1.2.13) EtOH 62% (m/m); (9) *Taraxacum*, Planta tota rec. Ø (Ph. Eur. 10/2535, V. 1.1.3).

All nine respective plants were organically cultivated in Germany on fields dedicated to medicinal plant cultivation.

For all experiments, dry extracts were prepared from the same batch of Amara Drops (Weleda AG, Schwäbisch Gmünd, Germany) and stored at room temperature until use. Dry extracts of individual herbal extracts entering in the composition of Amara extract were prepared from different batches of the same tinctures used to prepare Amara Drops.

Before experimental use, Amara extract and Amara individual extract stock solutions (hereafter referred to as “Amara extract” and “Amara individual extracts,” respectively) were prepared as detailed in the Supplementary Materials and Methods.

The herbal extract STW5 (Iberogast Classic, Bayer Vital GmbH, Leverkusen, Germany) was used as a control in some experiments.^28^, ^29^, ^30^, ^31^, ^32^ A STW5 lyophilisate was prepared and stored at room temperature until use. Before experimental use, STW5 extract stock solutions (hereafter referred to as “STW5 extract”) were prepared in the same conditions as for Amara extracts.

### 2.2 Composition analysis of Amara extract

The composition of Amara extract was evaluated by ultra‐high‐performance liquid chromatography with high‐resolution quadrupole time‐of‐flight tandem mass spectrometry (UHPLC‐hr‐QToF‐MS/MS), as described in the Supplementary Materials and Methods. Interpretation of the mass signals was conducted using Compass Data Analysis 4.2 and MetaboScape 5.0 software (Bruker, Billerica, MA, USA). Peak annotation was performed using the National Institute of Standards and Technology (NIST) Mass Spectral Library (U.S. Department of Commerce, Gaithersburg, MD, USA), and based on available literature citations.^1^, ^2^, ^3^, ^4^, ^5^, ^6^

### 2.3 Guinea pig fundus motility assays

Guinea pig fundus motility experiments were conducted by REPROCELL Europe (Glasgow, UK) under the license number XC2FD842E granted by the University of Glasgow Ethics Committee on 30 April 1987, amended on 26 September 2023, and approved by the User Research Ethics Board of the UK Home Office. Strips of circular muscle were dissected from the fundus of male adult Dunkin Hartley guinea pigs. Only tissues passing viability checks were used. Data are mean ± standard error of the mean (SEM) of at least three independent experiments, each performed using up to two animals. Depending on tissue availability per animal, several strips (or replicates) were used in each experiment, as indicated in the respective figure legends.

Fundus circular muscle strips of approximately 15 mm long and 2–3 mm wide were dissected from surrounding tissue and mucosa was removed. Fundus circular muscle strips were mounted in individual 25 mL organ baths containing physiological saline solution (PSS; 119 mM NaCl, 4.7 mM KCl, 1.2 mM MgSO_4_, 24.9 mM NaHCO_3_, 1.2 mM KH_2_PO_4_, 2.5 mM CaCl_2_, and 11.1 mM glucose) and maintained at 37°C under 95% O_2_/5% CO_2_ throughout the experiment. Changes in force production were recorded using transducers (TRI202PAD, Panlab Harvard Apparatus, Barcelona, Spain). After mounting in organ baths, the fundus muscle strips were equilibrated in PSS for 30 min before they were set to a stable tension of 1.0 g ± 0.2 g. Tissues were then allowed to equilibrate over 45 min with washes every 15 min, until stabilization of baseline tension.

Amara extract dose–response effects on smooth muscle relaxation and following carbachol‐induced fundus muscle contraction (with and without pretreatment with the M3 receptor antagonist J‐104129 [300 nM; Bio‐Techne, Abingdon, UK])^35^, ^36^ were measured as described in the Supplementary Materials and Methods. J‐104129 has a 120‐fold selectivity for M3 receptors (*K*_i_ = 4.2 nM) over M2 receptors ((*K*_i_ = 490 nM), and the applied concentration of 300 nM for J‐104129 is expected to strongly inhibit M3 without interfering with M2 receptor activity.^35^ Isoprenaline (100 pM–10 μM; Sigma‐Aldrich/Merck) was used as a smooth muscle relaxant control,^37^ and papaverine (100 μM; Sigma–Aldrich/Merck) was added after completion of each assay to induce complete smooth muscle relaxation and thus control for tissue viability.^27^, ^38^ Data were expressed as the percentage of change of the baseline tone response (smooth muscle relaxation experiment) or as the percentage of change of carbachol‐induced constriction (relaxation of carbachol‐induced muscle contraction). A control experiment verifying the inhibitory effect of the M3 receptor antagonist J‐104129 on carbachol‐induced contraction was performed as well, as described in the supplementary Materials and Methods.

### 2.4 M2 muscarinic receptor inhibition assay in CHO‐K1 recombinant cell line

The inhibitory effect of Amara extract on M2 muscarinic receptor activity was evaluated in CHO‐K1 cells expressing the recombinant human M2 receptor (accession number NP_000730.1) using the cAMP HTRF assay for Gi‐coupled receptors (FAST‐0261C; EuroscreenFast, Charleroi, Belgium), as described by the manufacturer and as detailed in the Supplementary Materials and Methods. Control cAMP HTRF assays for the Gi‐coupled receptors GPR35 (FAST‐0915C) and GPR84 (FAST‐0935C) were conducted for the M2 receptor, and as described in the supplementary Materials and Methods. Data were expressed as a percentage of inhibition of agonist‐induced receptor activation.

### 2.5 M3 muscarinic receptor inhibition assay in CHO‐K1 recombinant cell line

The inhibitory effect of Amara extract on M3 muscarinic receptor activity was evaluated in CHO‐K1‐mt aequorin cells expressing the recombinant human M3 receptor (accession number NP_000731.1) using the IPOne HTRF assay (ES‐212A; EuroscreenFast), as described by the manufacturer and as detailed in the Supplementary Materials and Methods. Dose–response data were expressed as a percentage of inhibition of acetylcholine‐induced M3 activation.

### 2.6 Cytotoxicity control assay

Cytotoxicity assays were conducted by EuroscreenFast in the CHO‐K1‐mt aequorin cell line expressing the human recombinant M3 receptor (see Section 2.5), as described in the Supplementary Materials and Methods, and data were expressed as percentage cytotoxicity relative to the vehicle control.

### 2.7 Radioligand M2 binding competition assay

Binding of Amara extract to the M2 muscarinic receptor was tested by radioligand binding competition assay (FAST‐0261B; EuroscreenFast), following the manufacturer's recommendations, using CHO‐K1 cell membrane extracts, as detailed in the Supplementary Materials and Methods. Data were expressed as the percentage of residual binding of the radiotracer or “control activator” to the M2 receptor.

### 2.8 Statistical analysis

All analyzed data were displayed graphically using GraphPad Prism version 9.2.0. (GraphPad Software Inc., San Diego, CA, USA). Statistical analysis of organ bath data was performed using the two‐way ANOVA with Dunnett's post‐hoc test in GraphPad Prism version 9.2.0., to compare the effect of Amara extract to that of the vehicle control, taking into account the variable number of replicates per condition. A *p*‐value of ≤0.05 was considered statistically significant. IC_50_ values of dose–response experiments (receptor activity and binding assays) were determined with the XLfit software version 5.5.0 (IDBS, Woking, UK) using nonlinear regression applied to a sigmoidal dose–response model (XL Fit fit Model 203). Cytotoxicity ≤20% relative to the vehicle control was not considered significant.

## 3 RESULTS

### 3.1 Amara extract composition

The composition of Amara extract was first evaluated by ultra‐high‐performance liquid chromatography with high‐resolution quadrupole time‐of‐flight tandem mass spectrometry (UHPLC‐hr‐QToF‐MS/MS). MS/MS analysis was conducted using electrospray ionization (ESI) in positive and negative modes. It allowed the annotation of 41 secondary metabolites in ESI positive mode (Figure S1A and Table S1) and 23 secondary metabolites in ESI negative mode (Figure S1B and Table S2).

Of the 23 analytes detected in negative ionization mode, 19 were also detected in positive ionization mode (Tables S1 and S2). A total of 31 unique secondary metabolites were identified. Of these, 16 (51.6%) were reported for their function in gastrointestinal protection, regulation, and/or motility, namely: apigenin,^39^ apigenin glucoside,^40^ apigenin glucuronide,^41^ caffeic acid ethyl ester,^42^ deoxylactucin,^43^ dicaffeoylquinic acid,^44^ genistein glucuronide,^39^ gentiopicrin (also known as gentiopicroside),^45^ imperatorin,^46^, ^47^ isoimperatorin,^47^ isorosmanol,^48^ nepetin glucoside (also known as nepitrin),^49^ rosmarinic acid,^50^ sweroside,^51^ swertiamarin,^52^ and tuberonic acid.^53^

Of the remaining 15 unique metabolites, 13 are known for their anti‐inflammatory, antioxidant, and/or antimicrobial activities, namely: apigenin glucopyranoside,^54^ cynarin,^55^ dimethyl rosmanol,^56^ hispidulin,^57^ hispidulin 7‐glucuronide,^57^ kaempferol rutinoside,^58^ luteolin,^59^ luteolin glucuronide,^59^ methyl catechin,^60^ mono caffeoylquinic acid,^61^ ostruthin,^62^ quercetin glucuronide,^63^ and rosmadial.^64^

Of the two remaining identified metabolites, pinusolide is a platelet‐activating factor antagonist^65^ with a hypoglycemic effect^66^ and no reported gastrointestinal, anti‐inflammatory, or anti‐oxidant activity, and tuberonic acid glucoside has—to the best of our knowledge—no reported function.

### 3.2 Amara extract induces fundus relaxation in an ex vivo animal model

**FIGURE 1:**
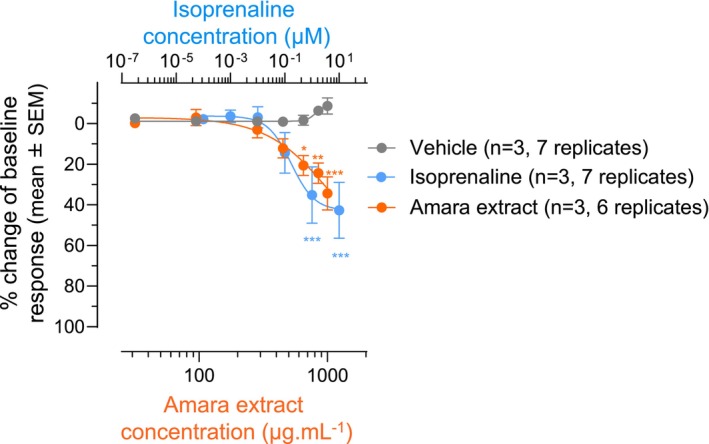
Amara extract induces fundus relaxation. Cumulative concentration response curves were conducted on fundus circular muscles isolated from guinea pigs. After stabilization of the baseline tone, muscle strips were exposed to either PSS (vehicle), isoprenaline (100 pM, 1 nM, 10 nM, 100 nM, 1 μM, and 10 μM) as a positive control for smooth muscle relaxation or Amara extract (31.5, 94.4, 283.3, 450, 650, 850, and 1000 μg mL^−1^) for a minimum of 20 min or until plateau of response. Changes in baseline tone were expressed as a percentage of change in the baseline tone response (mean ± SEM). Data are from three independent experiments (*n* = 3) using up to two animals per experiment and a total of 6 (Amara extract) or 7 (vehicle, isoprenaline) smooth muscle strips (defined as replicates). Two‐way ANOVA with Dunnet's post hoc test comparing test compounds (isoprenaline or Amara extract) to vehicle: *p < 0.05, **p < 0.01, ***p < 0.001.

The effect of Amara extract on fundus accommodation was investigated in an ex vivo animal model. Strips of circular muscle were dissected from the fundus of guinea pigs, and organ baths were used to measure changes in baseline muscle tone upon incubation with increasing concentrations of Amara extract. The smooth muscle relaxant isoprenaline was used as a positive control.^37^ Addition of increasing concentrations of both Amara extract and isoprenaline caused the relaxation of smooth muscle strips in a dose‐dependent manner (Figure 1). Changes in baseline tone compared to the vehicle control were statistically significant at the concentrations of 650, 850, and 1000 μg mL^−1^ Amara extract, and at 1 μM and 10 μM isoprenaline (Figure 1).

### 3.3 Amara extract inhibits carbachol‐induced contraction of fundus smooth muscles

Activation of the muscarinic G protein‐coupled receptors expressed in gastrointestinal smooth muscle cells, mainly M2 and M3 subtypes, is essential in controlling smooth muscle contraction and gastrointestinal motility.^67^ Accordingly, M2 and M3 muscarinic receptors of smooth muscle cells play a central role in the etiology of functional dyspepsia.^22^ Carbachol is known to induce smooth muscle contraction via activation of M2 and M3 receptors.^68^, ^69^ To investigate the possible implication of M2 and/or M3 receptors in Amara extract‐induced smooth muscle relaxation, we tested the ability of Amara extract to reverse carbachol‐induced fundus muscle contraction, using the ex vivo guinea pig fundus assay.

**FIGURE 2:**
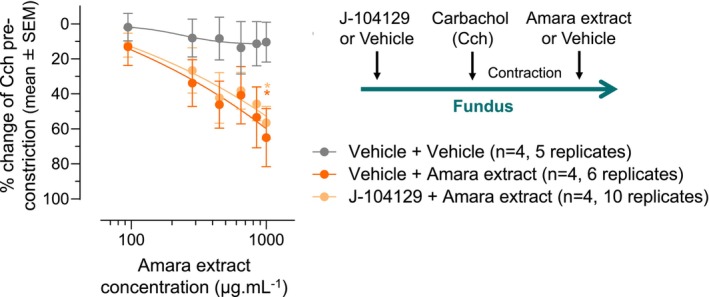
Amara extract‐mediated fundus relaxation is independent of M3 muscarinic receptor activity. Cumulative concentration response curves to Amara extract were conducted on fundus circular muscles from guinea pigs pretreated with the M3 receptor antagonist J‐104129 (300 nM) or 0.03% DMSO (vehicle). Contraction of the fundus muscle strips was induced by 10 μM carbachol. Muscle strips were then exposed to either PSS (vehicle) or Amara extract (concentrations 1–6, respectively: 94.4, 283.3, 450, 650, 850, and 1000 μg mL^−1^) for 10–15 min or until the plateau of response. Data are from four independent experiments (*n* = 4), each using up to two guinea pigs, and including a total of 5 (vehicle + vehicle), 6 (vehicle + Amara extract), or 10 (J‐104129 + Amara extract) strips (replicates). Results are expressed as a percentage of change of carbachol (Cch) constriction response (mean ± SEM). Two‐way ANOVA with Dunnet's post hoc test comparing Amara extract treatment (±J‐104129) to vehicle: *p < 0.05.

Addition of Amara extract on fundus muscle strips precontracted by treatment with carbachol reduced carbachol‐induced muscle contraction in a dose‐dependent manner (Figure 2, dark orange curve). Compared to the vehicle control, Amara extract's relaxation effect was statistically significant at the concentration of 1000 μg mL^−1^. This result indicates that the Amara extract can inhibit carbachol‐induced smooth muscle contraction, possibly via the inhibition of the muscarinic receptors M2 and/or M3.

### 3.4 Amara extract's relaxant effect does not depend on M3 muscarinic receptor

To identify the muscarinic receptors involved in Amara extract's relaxant effect, the ex vivo guinea pig fundus assay was repeated following treatment with J‐104129, an M3 muscarinic receptor antagonist with a high selectivity for M3 over M2 receptors.^35^, ^36^ The efficacy of the M3 antagonist J‐104129 on carbachol‐induced contraction was first verified in the organ bath model. Pretreatment of the fundus smooth muscle strips with 300 nMJ‐104129 resulted in a significant reduction of carbachol‐induced muscle contraction (Figure S2). As expected from the inhibition of M3 but not M2 receptors (at the J‐104129 concentration of 300 nM), the relaxation effect of J‐104129 was partial (Figure S2). This allowed us to investigate the relaxation effect of Amara extract on the remaining (mainly M2‐mediated) carbachol‐induced constriction of fundus strips pretreated with J‐104129 versus the vehicle control (Figure 2). Pretreatment of the fundus smooth muscle strips with J‐104129 did not affect the relaxation effect of Amara extract on carbachol‐induced fundus contraction (Figure 2, light orange curve) compared to the vehicle control (Figure 2, dark orange curve). Since carbachol induces smooth muscle contraction via M2 and M3 receptors,^68^, ^69^ this result suggests that the relaxant effect of Amara extract is M3‐independent and likely involves the M2 receptor.

**FIGURE 3:**
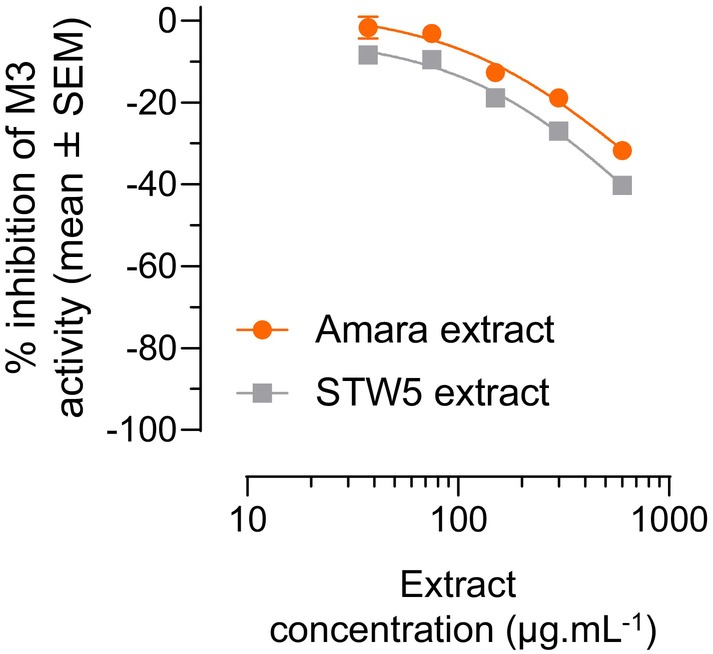
Amara extract does not inhibit M3 muscarinic receptor activity. CHO‐K1‐mt aequorin cells expressing the recombinant human M3 receptor were pretreated with Amara extract or STW5 extract (38, 75, 150, 300, and 600 μg mL^−1^) and treated with the M3 agonist acetylcholine. M3 activity was assessed using the IPOne HTRF assay, and the percentage of M3 activity inhibition was measured at each extract concentration (mean ± SEM). Neither Amara extract nor STW5 extract inhibited acetylcholine‐induced M3 activity.

To verify whether the relaxant effect of Amara extract detected on guinea pig fundus is independent of the M3 receptor, we assessed the dose‐dependent effect of Amara extract on M3 activation in CHO‐K1 cells expressing the recombinant human M3 receptor. M3‐expressing cells were pretreated with increasing concentrations of Amara extract and activated by the M3 agonist acetylcholine. A working solution prepared from lyophilized STW5 (STW5 extract) and used in the same experimental conditions as the Amara extract was tested in parallel, as a control herbal extract with known fundus smooth muscle relaxant activity.^28^, ^29^, ^30^, ^31^, ^32^ Neither Amara extract nor STW5 extract inhibited acetylcholine‐induced M3 receptor activity (Figure 3). This observation confirms that the relaxant effect of Amara extract is M3 independent.

### 3.5 Amara extract inhibits the activity of the M2 muscarinic receptor

**FIGURE 4:**
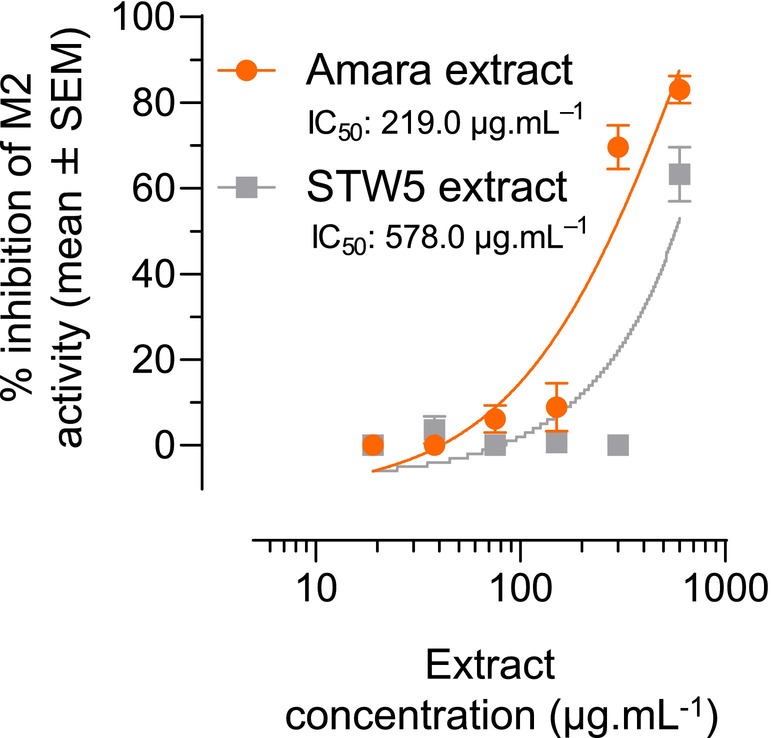
Amara extract inhibits M2 muscarinic receptor activity. CHO‐K1 cells expressing the recombinant human M2 receptor were pretreated with Amara extract or STW5 extract (9, 19, 38, 75, 150, 300, and 600 μg mL^−1^) and then treated with forskolin and the M2 agonist oxotremorine. M2 activity was assessed using the cAMP HTRF assay for Gi‐coupled receptor, and the percentage of M2 activity inhibition was measured at each extract concentration (mean ± SEM). A significant inhibition of oxotremorine‐induced M2 activity was observed for both Amara extract and STW5 extract.

To further investigate whether the relaxant effect of Amara extract detected on guinea pig fundus smooth muscles is mediated by the M2 muscarinic receptor, we assessed the dose‐dependent effect of Amara extract on M2 activation in CHO‐K1 cells expressing the recombinant human M2 receptor. Cells were pretreated with increasing concentrations of Amara extract and activated by the M2 agonist oxotremorine in the presence of forskolin. Amara extract strongly inhibited oxotremorine‐induced M2 activity and in a dose‐dependent manner (IC_50_ 219.0 μg mL^−1^) (Figure 4, orange curve). STW5 extract tested in parallel also inhibited M2 activity, with an IC_50_ of 578.0 μg mL^−1^ (Figure 4, gray curve). These data strongly suggest that the relaxant effect of Amara extract on carbachol‐induced muscle contraction is mediated by the M2 muscarinic receptor.

### 3.6 Three of the nine herbal components of Amara extract inhibit M2 receptor activity

**FIGURE 5:**
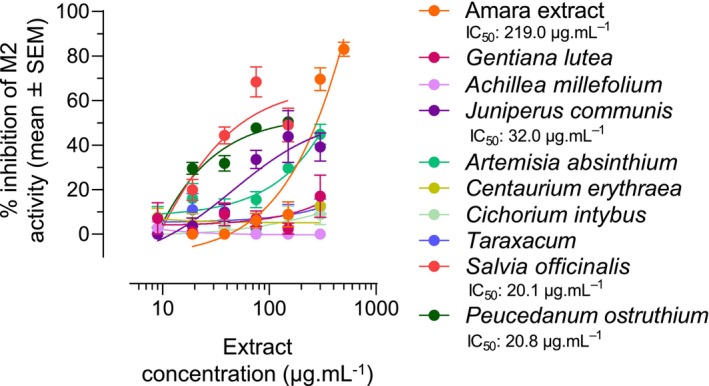
Several components of Amara extract inhibit M2 muscarinic receptor activity. CHO‐K1 cells expressing the recombinant human M2 receptor were pretreated with Amara extract (9, 19, 38, 75, 150, 300, and 600 μg mL^−1^) or with Amara individual extracts (9, 19, 38, 75, 150, and 300 μg mL^−1^) then treated with forskolin and the M2 agonist oxotremorine. M2 activity was assessed using the cAMP HTRF assay for Gi‐coupled receptor, and the percentage of M2 activity inhibition was measured at each extract concentration (mean ± SEM). A significant inhibition of oxotremorine‐induced M2 activity was observed for Amara extract and three of the nine Amara individual extracts: *Juniperus communis*, *Peucedanum ostruthium*, and *Salvia officinalis*.

We next investigated whether any of the nine herbal components of Amara extract were active in inhibiting M2 receptor activity in CHO‐K1 cells. CHO‐K1 cells were pretreated with increasing concentrations (9–300 μg mL^−1^) of the nine Amara individual extracts and activated with the M2 receptor agonist, as before. Three of the nine herbal extracts showed an inhibitory effect on M2 activity, namely: *Juniperus communis* (IC_50_ 32.0 μg mL^−1^), *P. ostruthium* (IC_50_ 20.8 μg mL^−1^), and *Salvia officinalis* (IC_50_ 20.1 μg mL^−1^) (Figure 5).

A control experiment showed that Amara extracts' inhibitory effect on M2 activity in CHO‐K1 cells and that of the three active components were not due to cytotoxicity, at least at their respective IC_50_ (Figure S3). *P*. *ostruthium* showed some cytotoxicity at concentrations of 150 and 300 μg mL^−1^, thus well above its IC_50_ of 20.8 μg mL^−1^.

We also verified that the inhibitory effect of Amara extract was specific for the M2 muscarinic receptor, that is, did not target any G protein‐coupled receptor, by evaluating its impact on the activity of GPR35 and GPR84. GPR35 and GPR84 are members of the G protein‐coupled receptor family with a known function in intestinal health and disease.^70^, ^71^ Treatment of CHO‐K1 cells expressing GPR35 or GPR84 with a high concentration of Amara extract (500 μg mL^−1^) caused a limited (≤30.1%) inhibition of agonist‐induced receptor activity, compared to 94.8% inhibition of the M2 receptor by the same concentration of Amara extract (Figure S4).

### 3.7 Amara extract binds the M2 muscarinic receptor

**FIGURE 6:**
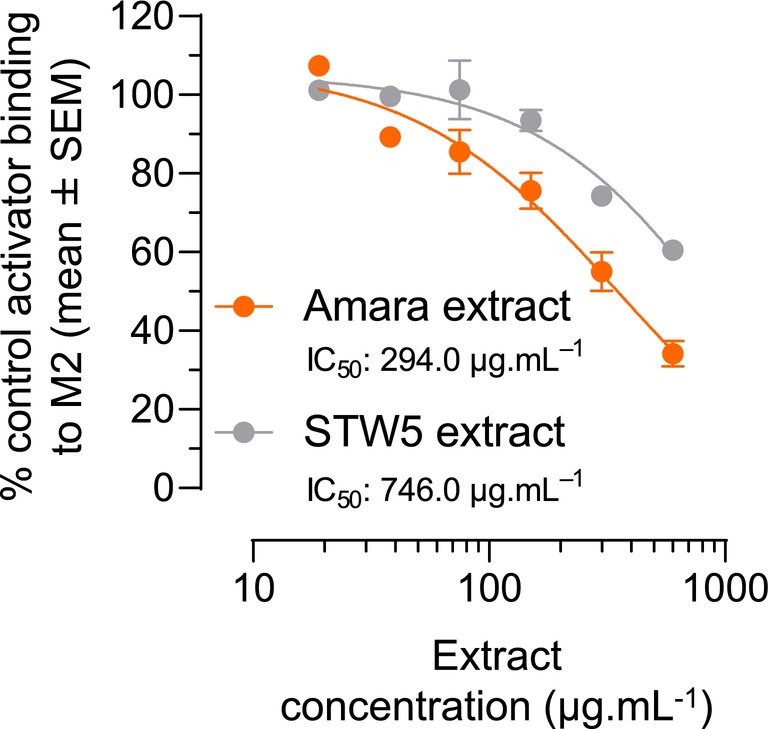
Amara extract binds the M2 muscarinic receptor. Radioligand binding competition assay was performed using membrane extracts of CHO‐K1 cells in the presence of the radiolabelled M2 receptor ligand [^3^H] N‐methylscopolamine (“Control Activator”) and increasing concentrations (19, 38, 75, 150, 300, and 600 μg mL^−1^) of Amara extract or STW5 extract. Results are expressed as the percentage of residual binding of labeled Control Activator to the M2 receptor for each concentration of herbal extract (mean ± SEM).

We further assessed whether Amara extract's inhibitory effect on M2 activity involved its binding to the M2 receptor. Radioligand binding competition assays were conducted using membrane extracts of CHO‐K1 cells (expressing the M2 receptor) in the presence of the radiolabelled ligand N‐methylscopolamine and increasing concentrations of Amara extract or STW5 extract. Both Amara extract and STW5 extract specifically bound the M2 receptor, with an IC_50_ of 294.0 and 746.0 μg mL^−1^, respectively (Figure 6).

**FIGURE 7:**
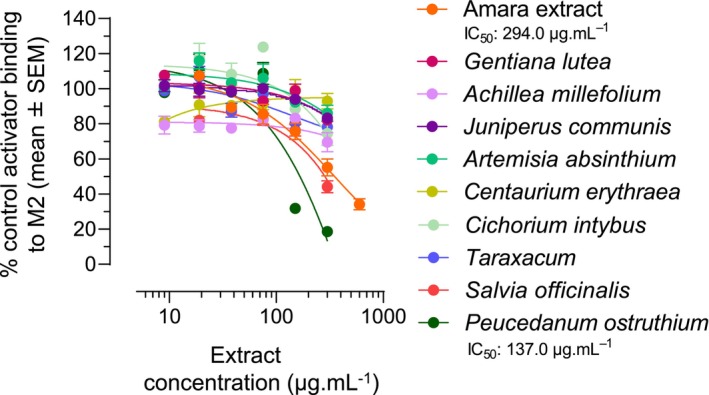
Some components of Amara extract bind the M2 muscarinic receptor. Radioligand binding competition assay was performed as in Figure 6, using increasing concentrations (9, 19, 38, 75, 150, and 300 μg mL^−1^) of Amara individual extracts. Results are expressed as the percentage of residual binding of labeled Control Activator to the M2 receptor for each concentration of herbal extract (mean ± SEM). *Peucedanum ostruthium* bound the M2 receptor with an IC_50_ of 137.0 μg mL^−1^.

To investigate whether any of the nine herbal components of Amara extract were active in binding the M2 receptor, radioligand binding competition assays were repeated using increasing concentrations (9–300 μg mL^−1^) of the nine Amara individual extracts. *P. ostruthium* (IC_50_ 137.0 μg mL^−1^) and to a lesser extent *Salvia officinalis* (no calculable IC_50_) demonstrated binding to the M2 receptor (Figure 7).

### 3.8 *Peucedanum ostruthium* inhibits carbachol‐induced fundus muscle contraction

To test the potential fundus relaxation effect of the Amara individual extracts that showed some inhibitory or binding activity toward the M2 receptor (i.e., *P. ostruthium, Salvia officinalis*, and *Juniperus communis*), we tested their ability to inhibit carbachol‐induced fundus muscle contraction in the ex vivo guinea pig fundus assay. As described above (Figure 2), organ baths were pretreated with the M3 antagonist J‐104129 or vehicle prior to muscle contraction by carbachol.

**FIGURE 8:**
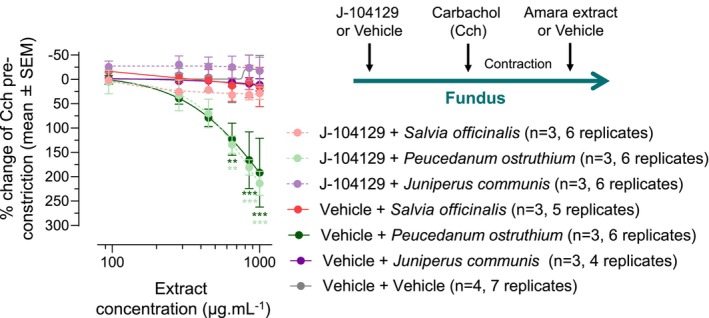
*Peucedanum ostruthium* can reverse carbachol‐induced fundus smooth muscle contraction, in an M2‐dependent manner. Cumulative concentration response curves to Amara individual extracts of *P. ostruthium*, *Salvia officinalis*, and *Juniperus communis* were conducted on fundus circular muscles from guinea pigs pretreated with the M3 receptor antagonist J‐104129 (300 nM) or 0.03% DMSO (vehicle). Contraction of the fundus muscle strips was induced by 10 μM carbachol. Muscle strips were then exposed to either PSS (vehicle) or herbal extracts (94.4, 283.3, 450, 650, 850, and 1000 μg mL^−1^) for 10–15 min or until plateau of response. Data are from three (*n* = 3) or four (*n* = 4) independent experiments, each conducted with up to two guinea pigs, including a total of 4–7 strips (replicates). Results are expressed as a percentage of change of carbachol (Cch) constriction response (mean ± SEM). Two‐way ANOVA with Dunnet's post hoc test comparing Amara individual extract treatment (±J‐104129) to vehicle: **p < 0.01, ***p < 0.001.

Addition of increasing concentrations (94.4–1000 μg mL^−1^) of *P. ostruthium*, but not of *Salvia officinalis* or *Juniperus communis*, reduced carbachol‐induced muscle contraction in a dose‐dependent manner and regardless of the presence of J‐104129 (Figure 8, red curves). Compared to the vehicle control, *P. ostruthium*'s relaxation effect was statistically significant at the concentration of 650, 850, and 1000 μg mL^−1^. These results indicate that Amara's herbal component *P. ostruthium* exerts a relaxation effect on fundus smooth muscles, likely via binding and inhibition of the M2 muscarinic receptor.

## 4 DISCUSSION

This experimental study demonstrated that Amara extract induces fundus relaxation and reduces carbachol‐induced contraction of guinea pig fundus smooth muscle strips in a dose‐dependent manner. This smooth muscle relaxant effect likely involves the M2 but not the M3 muscarinic G protein‐coupled receptor. Amara extract specifically inhibited and bound the M2 receptor in CHO‐K1 cells, with IC_50_ in a comparable range (219 μg mL^−1^ and 294 μg mL^−1^, respectively). Our data thus strongly suggest that Amara extract induces smooth muscle relaxation by directly inhibiting M2 muscarinic receptor response. This proposition is supported by the previous demonstration that the prokinetic agent acotiamide, which targets muscarinic receptors, improves gastric motility in patients with impaired gastric accommodation.^21^, ^24^ Interestingly, at the recommended dosage of Amara Drops (15 drops or 348 μL),^7^ the amount of Amara dry extract ingested would equal 3.97 mg (see Section 2.1). Considering an average gastric volume of 10 mL for an empty stomach,^72^, ^73^ the final concentration of Amara dry extract would be about 397 μg mL^−1^, hence in range with its effective concentration as a regulator of M2 activity. If the relaxant effect of Amara extract on fundus smooth muscle observed in the guinea pig animal model is confirmed in humans, our results suggest a potential benefit of Amara Drops in the management of gastric accommodation.

Of the nine herbal components of Amara Drops, *Juniperus communis*, *P. ostruthium*, and *Salvia officinalis* inhibited M2 receptor activity with an IC_50_ of 32.0, 20.8, and 20.1 μg mL^−1^, respectively. No cytotoxicity was associated with these extracts at these concentrations. The concentrations of 150 and 300 μg mL^−1^ at which *P. ostruthium* exhibited some cytotoxicity were well above its IC_50_ value of 20.8 μg mL^−1^. In addition to inhibiting M2 receptor activity, *P. ostruthium*—and to some extent *Salvia officinalis*—bound the M2 receptor. Calculated IC_50_ of binding of *P. ostruthium* to M2 using CHO‐K1 membrane fractions was 137 μg.mL^−1^. Given the cytotoxicity effect noted for *P. ostruthium* in CHO‐K1 cells at concentrations of 150 μg mL^−1^ and higher, one should take this binding result with caution. Indeed, one cannot exclude at this stage the presence of precipitates in our functional and binding assays at high concentrations of *P. ostruthium* extract, although none were observed by eye, which could explain the associated cytotoxicity and possibly result in some non‐specific receptor binding. Further studies should clarify this possibility and confirm our results. Finally, *P. ostruthium* extract also reduced carbachol‐induced smooth muscle contractions at concentrations ≥283.3 μg mL^−1^ in a dose‐dependent manner. No apparent toxicity was noted in these organ‐bath experiments, as evidenced by the post‐assay viability control (papaverine‐induced muscle relaxation).

The comparison of the IC_50_ values for M2 inhibition obtained with Amara extract to those of active individual extracts reveals some differences. At a concentration of Amara extract of 219.0 μg mL^−1^ (its IC_50_ for M2 inhibition), the concentration of active individual extracts is expected to equal 2.0 μg mL^−1^ for *Juniperus communis*, 7.0 μg mL^−1^ for *P. ostruthium*, and 50.3 μg mL^−1^ for *Salvia officinalis*. These concentrations are 16 times lower, 3 times lower, and 2.5 times higher, respectively, than those of their IC_50_ when tested as individual extracts (i.e., 32.0, 20.8, and 20.1 μg mL^−1^, respectively). While these concentration ranges are reasonably close for *P. ostruthium* and *Salvia officinalis*, they are quite apart for *Juniperus communis*, suggesting that the latter is unlikely to be an active M2 inhibitor within Amara extract. This is also true when considering our estimation of gastric concentration for the recommended Amara Drops dosage described above (397 μg mL^−1^ Amara extract). In that case, *Juniperus communis* concentration would still lie 8.6 times below its IC_50_ for M2 inhibition (3.7 vs. 32.0 μg mL^−1^), while *P. ostruthium* and *Salvia officinalis* concentrations would be in a similar range (12.7 vs. 20.8 μg mL^−1^ for *P. ostruthium* and 91.1 vs. 20.1 μg mL^−1^ for *Salvia officinalis*). A potential implication of *P. ostruthium* and *Salvia officinalis* in regulating M2 function is further supported by their substantial M2 binding activity. Intriguingly, *P. ostruthium* but not *Salvia officinalis* was active in reversing carbachol‐induced fundus contraction in the organ bath model. The reason for this discrepancy is unclear. It might suggest that M2 receptor inhibition is necessary but not sufficient to mediate muscle relaxation. Our results might indicate that despite a role in binding and inhibiting M2 muscarinic receptor, *Salvia officinalis* might require the cooperation of other herbal components within Amara extract to mediate muscle relaxation. Further studies should assess this question, by investigating the potential synergy between Amara individual extracts. Altogether, our data suggest a role of (at least) *P. ostruthium* and *Salvia officinalis* (possibly in cooperation with other herbal components) in regulating M2 muscarinic receptor function and controlling fundus relaxation.

The three herbal extracts *Juniperus communis*, *P. ostruthium*, and *Salvia officinalis* that showed some inhibitory effect on M2 activity are rich in glycosidic derivatives (e.g., flavonoid glycosides, saponins), phenolic compounds (e.g., coumarins, flavonoids, caffeic acid, 3‐Caffeoylquinic acid), terpenes, and furocoumarins (e.g., imperatorin),^74^, ^75^, ^76^, ^77^, ^78^ some of which are associated with gastrointestinal protection, regulation, and/or motility (Tables S1 and S2). This suggests that some of these compounds might be responsible for the relaxant effect of Amara extract via regulation of M2 muscarinic receptor function. Future studies should also focus on identifying the active secondary metabolites involved in that process.

We found that, like for Amara extract, an extract prepared from lyophilized STW5 inhibited M2 but not M3 receptor activity, and that it bound the M2 receptor (IC_50_ for M2 inhibition and M2 binding of 578 and 746 μg mL^−1^, respectively). STW5 is a well‐described herbal preparation with a relaxant effect on gastrointestinal motility.^28^, ^31^, ^32^, ^33^ It is composed of nine plant extracts (*Chelidonium majus* L., *Mentha piperita* L., *Carum carvi* L., *Glycyrrhiza glabra* L., *Iberis amara* L., *Matricaria recutita* L., *Silybum marianum* L., *Melissa officinalis*, and *Angelica archangelica* L.) distinct from those composing the Amara extract (*Artemisia absinthium*, *Centaurium erythraea*, *Cichorium intybus*, *Gentiana lutea*, *Juniperus communis*, *Achillea millefolium*, *P. ostruthium*, *Salvia officinalis*, and *Taraxacum*). However, STW5 contains similar types of substances to those identified in Amara extract, in particular flavonoids, coumarins, terpenes, phenolic compounds, saponins, and furocoumarins,^32^ which might explain their similar activity profile, notably on fundus smooth muscle relaxation. Previous studies showed that STW5 binds the M3 muscarinic receptor.^22^ To the best of our knowledge, the effect of STW5 extract on M3 receptor activity has not been reported yet, nor has its effect on M2 receptor binding and activity. The present study now indicates that an STW5 extract prepared from lyophilized STW5 does not affect the activity of M3, despite a reported binding interaction, and that it binds and inhibits M2 receptor activity, similar to Amara extract. These observations further emphasize the relevance and potential benefit of herbal extracts such as Amara Drops in controlling gastric accommodation via regulating M2 muscarinic receptor function.

In conclusion, this study demonstrates the relaxant effect of Amara extract on fundus smooth muscles in a guinea pig muscle strip organ bath model, supporting its potential benefit in the prevention and/or treatment of impaired gastric accommodation. It also suggests the implication of the M2 muscarinic receptor in mediating that effect, thus providing novel insights into Amara extract's mode of action. Additional pharmacological, animal, and human studies are needed to further investigate the mechanism of action of Amara extract and its potential benefit for the management of gastric accommodation in patients with functional dyspepsia.

## AUTHOR CONTRIBUTIONS

M.‐R.P.‐B. and G.K. designed the study. M.‐R.P.‐B. administered the study, analyzed and interpreted the data, drafted the figures, and contributed to drafting the manuscript. G.K. supervised the study. J.R. contributed to designing the study. All authors critically reviewed and approved the manuscript.

## FUNDING INFORMATION

This research received no external funding.

## CONFLICT OF INTEREST STATEMENT

M.‐R.P.B., J.R., and G.K. are employees of Weleda AG. This preclinical study was funded by Weleda AG. The funder was involved in the choice of the research project, the design, and execution of the study, in data interpretation, in the decision to publish the results, and in the writing of the manuscript.

## Supporting information


**Figure S1.** Representative base peak chromatograms generated through UHPLC‐hr‐QtoF‐MS/MS analysis in electrospray ionization positive mode (A) and negative mode (B). Analyte annotation was performed based on literature^1^, ^2^, ^3^, ^4^, ^5^, ^6^ and database information. The list of analytes according to their retention time is shown in Table S1 (positive ion mode) and Table S2 (negative ion mode).
**Figure S2.** Control experiment verifying the relaxation effect of the M3 antagonist J‐104129 on carbachol‐induced contraction of guinea pigs’ fundus smooth muscle strips. (A) Effect of vehicle (DMSO) on carbachol constriction response of fundus circular smooth muscle strips isolated from guinea pigs. Muscle strips were incubated with DMSO (0.03%) between two exposures with carbachol (10 μM). Data are expressed as gram (g) tension and are the mean (±SEM) of seven independent experiments, each conducted on muscle strips dissected from two animals (50 strips or replicates per condition). (B) Effect of J‐104129 on carbachol constriction response of fundus circular smooth muscle strips isolated from guinea pigs. Muscle strips were incubated with J‐104129 (300 nM) between two exposures with carbachol (10 μM). Data are expressed as gram (g) tension and are the mean (±SEM) of six independent experiments, each conducted on muscle strips dissected from two animals (26 strips or replicates per condition).**p < 0.01.
**Figure S3.** Cytotoxicity of Amara extract and Amara individual extracts in CHO‐K1 cells. CHO‐K1‐mt aequorin cells were treated with Amara extract or STW5 extract (100, 300, 600, and 850 μg mL^−1^) or with Amara individual extracts (9, 19, 38, 75, 150, and 300 μg mL^−1^) for 24 h at 37°C under 5% CO_2_. Cytotoxicity was measured using the CellTiter 96® AQueous One Solution Cell Proliferation Assay, and data were expressed as % cytotoxicity relative to the vehicle control. *Peucedanum ostruthium* showed some cytotoxicity at the highest concentrations of 150 and 300 μg mL^−1^.
**Figure S4.** Amara extract does not inhibit the activity of the G protein‐coupled receptors GPR35 and GPR84. CHO‐K1 cells expressing the recombinant human M2, GPR35 or GPR84 receptor were pre‐treated with Amara extract (500 μg mL^−1^) or vehicle (0.1% ethanol) and activated by the respective agonist (oxotremorine for M2, zaprinast for GPR35 and capric acid for GPR84) at their EC_80_. Receptor activity was assessed using the cAMP HTRF assay for Gi‐coupled receptors, and results were expressed as the % of inhibition of the reference agonist activity. Amara extract inhibited the M2 but not the GPR35 or GPR84 receptor activity.

**Table S1.** Peak intensity of the analytes recorded at the indicated retention times (RT) in electrospray ionization positive mode through UHPLC‐hr‐QToF‐MS/MS analysis (see also **Figure** **S1** **A**).
Table S2. Peak intensity of the analytes recorded at the indicated retention times (RT) in electrospray ionization negative mode through UHPLC‐hr‐QtoF‐MS/MS analysis (see also **Figure** **S1** **B**).

**Data S1:** Supporting Information.

## Data Availability

The data presented in this study are available in the article and Supplementary Materials.
