# Supplementary material for: Herbal Amara extract induces gastric fundus relaxation via inhibition of the M2 muscarinic receptor
Source: Neurogastroenterol Motil. 2024 Sep 30;37(1):e14924. doi: 10.1111/nmo.14924 (PMC11650409; doi:10.1111/nmo.14924)
Supplement: Supplementary file 1 — Figure S1. Representative base peak chromatograms generated through UHPLC‐hr‐QtoF‐MS/MS analysis in electrospray ionization positive mode (A) and negative mode (B). Analyte annotation was performed based on literature 1 , 2 , 3 , 4 , 5 , 6 and database information. The list of analytes according to their retention time is shown in Table S1 (positive ion mode) and Table S2 (negative ion mode). Figure S2. Control experiment verifying the relaxation effect of the M3 antagonist J‐104129 on carbachol‐induced contraction of guinea pigs’ fundus smooth muscle strips. (A) Effect of vehicle (DMSO) on carbachol constriction response of fundus circular smooth muscle strips isolated from guinea pigs. Muscle strips were incubated with DMSO (0.03%) between two exposures with carbachol (10 μM). Data are expressed as gram (g) tension and are the mean (±SEM) of seven independent experiments, each conducted on muscle strips dissected from two animals (50 strips or replicates per condition). (B) Effect of J‐104129 on carbachol constriction response of fundus circular smooth muscle strips isolated from guinea pigs. Muscle strips were incubated with J‐104129 (300 nM) between two exposures with carbachol (10 μM). Data are expressed as gram (g) tension and are the mean (±SEM) of six independent experiments, each conducted on muscle strips dissected from two animals (26 strips or replicates per condition).**p < 0.01. Figure S3. Cytotoxicity of Amara extract and Amara individual extracts in CHO‐K1 cells. CHO‐K1‐mt aequorin cells were treated with Amara extract or STW5 extract (100, 300, 600, and 850 μg mL−1) or with Amara individual extracts (9, 19, 38, 75, 150, and 300 μg mL−1) for 24 h at 37°C under 5% CO2. Cytotoxicity was measured using the CellTiter 96® AQueous One Solution Cell Proliferation Assay, and data were expressed as % cytotoxicity relative to the vehicle control. Peucedanum ostruthium showed some cytotoxicity at the highest concentrations of 150 and 300 μg mL−1. Figure S4. Ama [file NMO-37-e14924-s003.pdf]

A

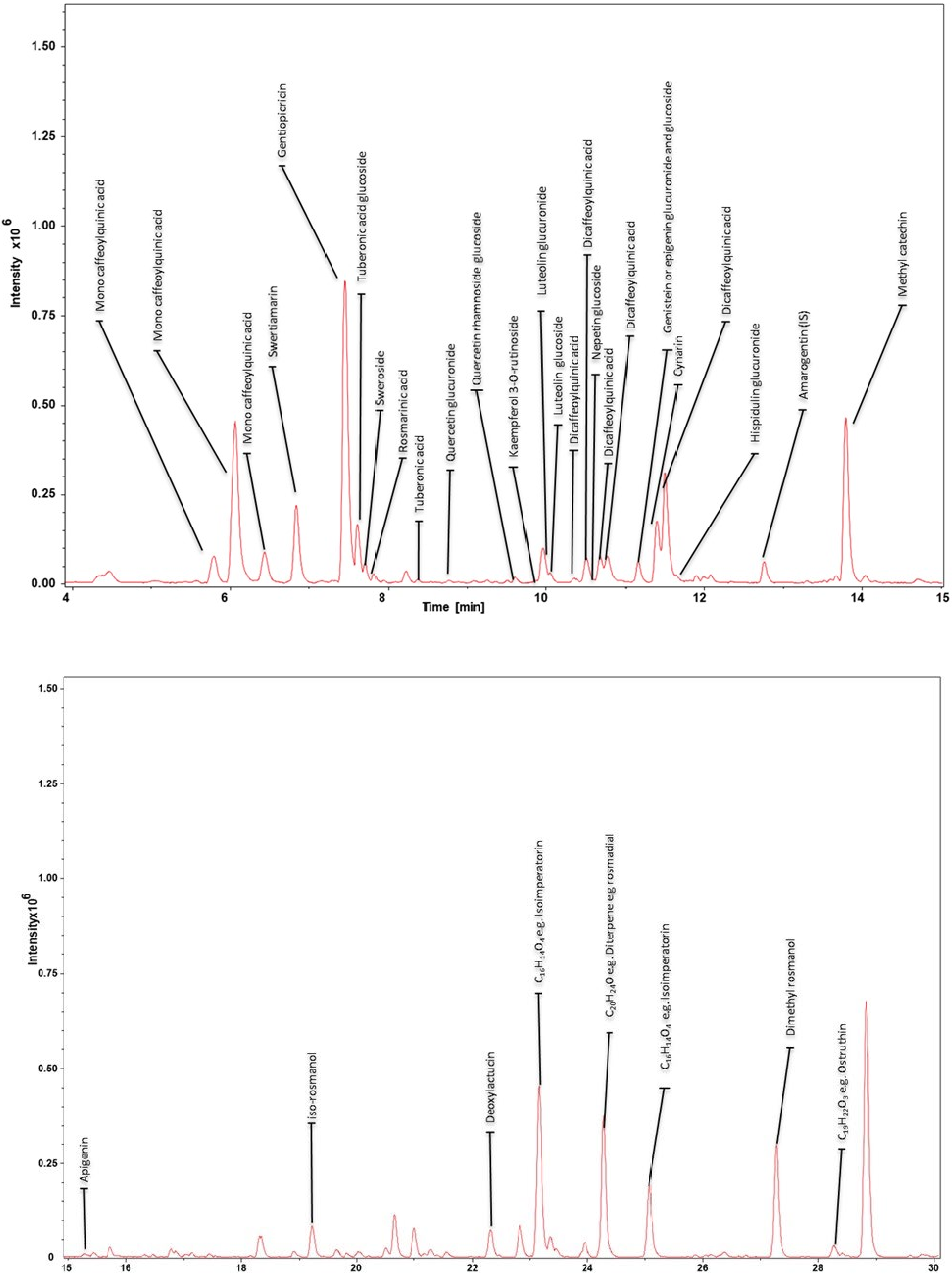
Intensity x10^6
Time [min]
Mono caffeoylquinic acid
Mono caffeoylquinic acid
Mono caffeoylquinic acid
Swertiamarin
Gentiopicricin
Tuberonic acid glucoside
Sweroside
Rosmarinic acid
Tuberonic acid
Quercetin glucuronide
Quercetin rhamnoside glucoside
Kaempferol 3-O-rutinoside
Luteolin glucuronide
Luteolin glucoside
Dicaffeoylquinic acid
Dicaffeoylquinic acid
Nepetin glucoside
Dicaffeoylquinic acid
Dicaffeoylquinic acid
Genistein or epigenin glucuronide and glucoside
Cynarin
Dicaffeoylquinic acid
Hispidulin glucuronide
Amarogentin (IS)
Methyl catechin
Intensityx10^6
Apigenin
iso-rosmanol
Deoxylactucin
$C_{16}H_{14}O_4$ e.g. Isoimperatorin
$C_{20}H_{24}O$ e.g. Diterpene e.g rosmadial
$C_{16}H_{14}O_4$ e.g. Isoimperatorin
Dimethyl rosmanol
$C_{19}H_{22}O_3$ e.g. Ostruthin

B

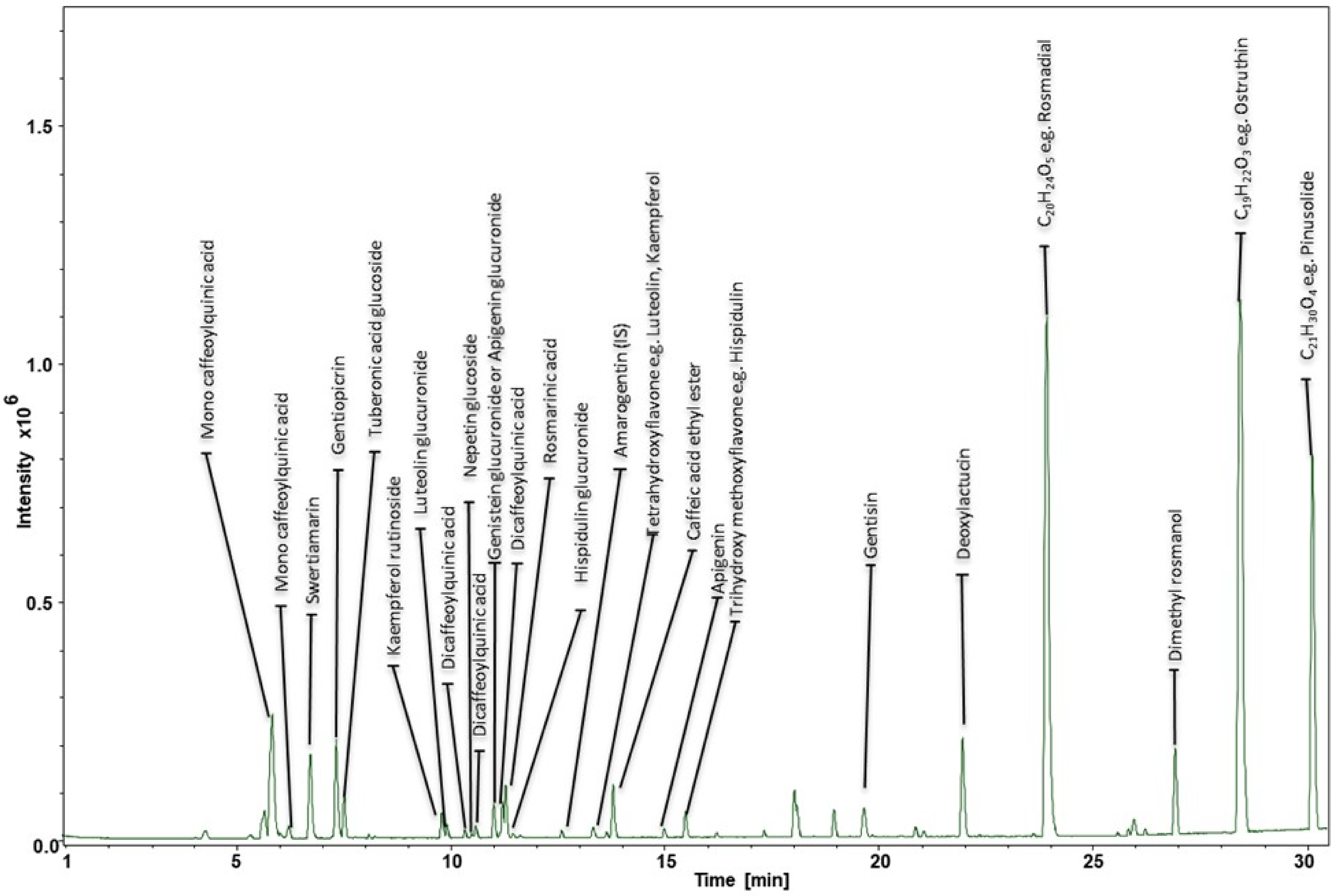

Figure S1. Representative base peak chromatograms generated through UHPLC-hr-QToF-MS/MS analysis in electrospray ionization positive mode (A) and negative mode (B). Analyte annotation was performed based on literature (references 29–34) and database information. The list of analytes according to their retention time is shown in Table S1 (positive ion mode) and Table S2 (negative ion mode).

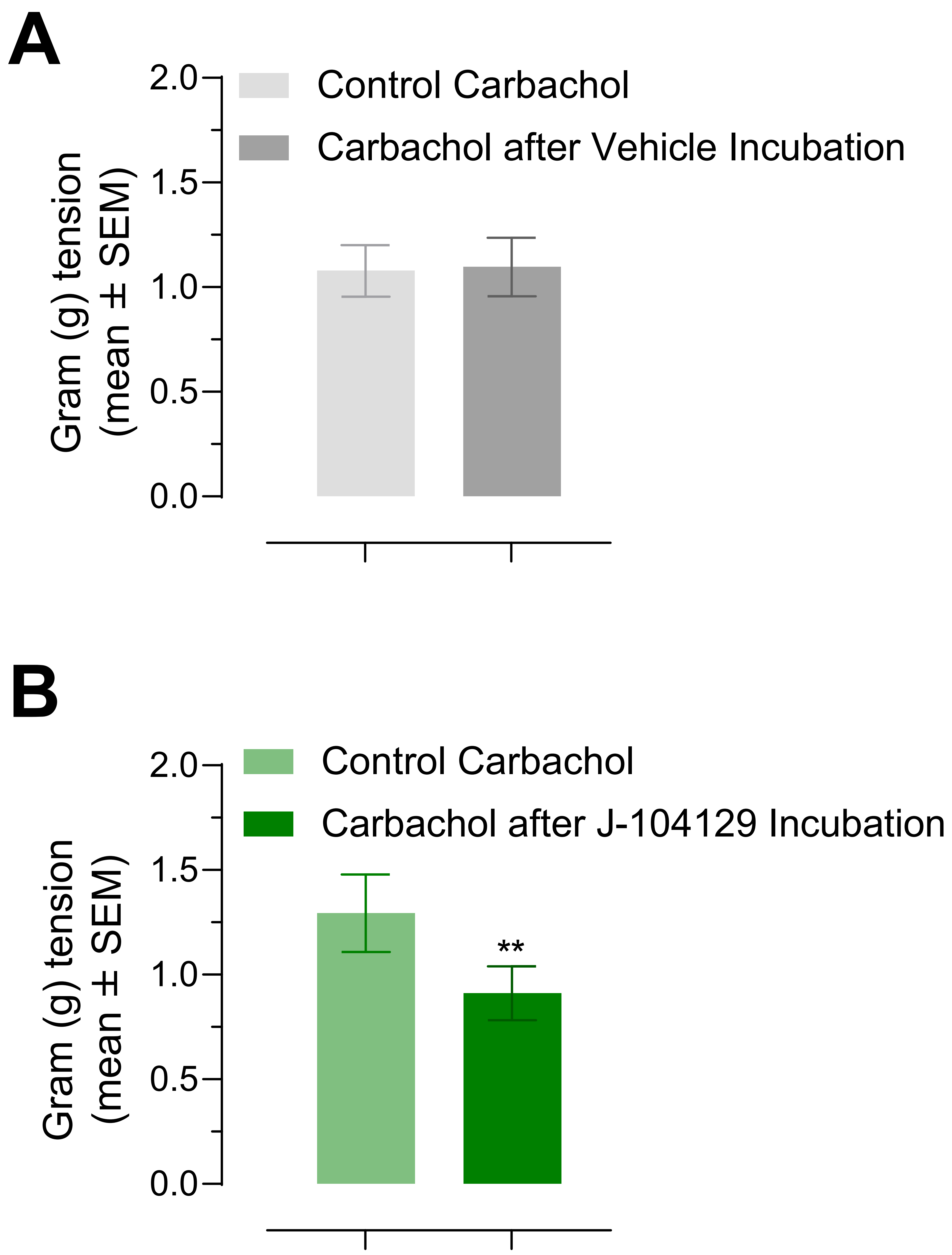

Figure S2. Control experiment verifying the relaxation effect of the M3 antagonist J-104129 on carbachol-induced contraction of guinea pigs' fundus smooth muscle strips. (A) Effect of vehicle (DMSO) on carbachol constriction response of fundus circular smooth muscle strips isolated from guinea pigs. Muscle strips were incubated with DMSO (0.03%) between two exposures with carbachol (10 μM). Data are expressed as gram (g) tension and are the mean (±SEM) of 7 independent experiments, each conducted on muscle strips dissected from 2 animals (50 strips or replicates per condition). **(B)** Effect of J-104129 on carbachol constriction response of fundus circular smooth muscle strips isolated from guinea pigs. Muscle strips were incubated with J-104129 (300 nM) between two exposures with carbachol (10 μM). Data are expressed as gram (g) tension and are the mean (±SEM) of 6 independent experiments, each conducted on muscle strips dissected from 2 animals (26 strips or replicates per condition).**$p<0.01$.

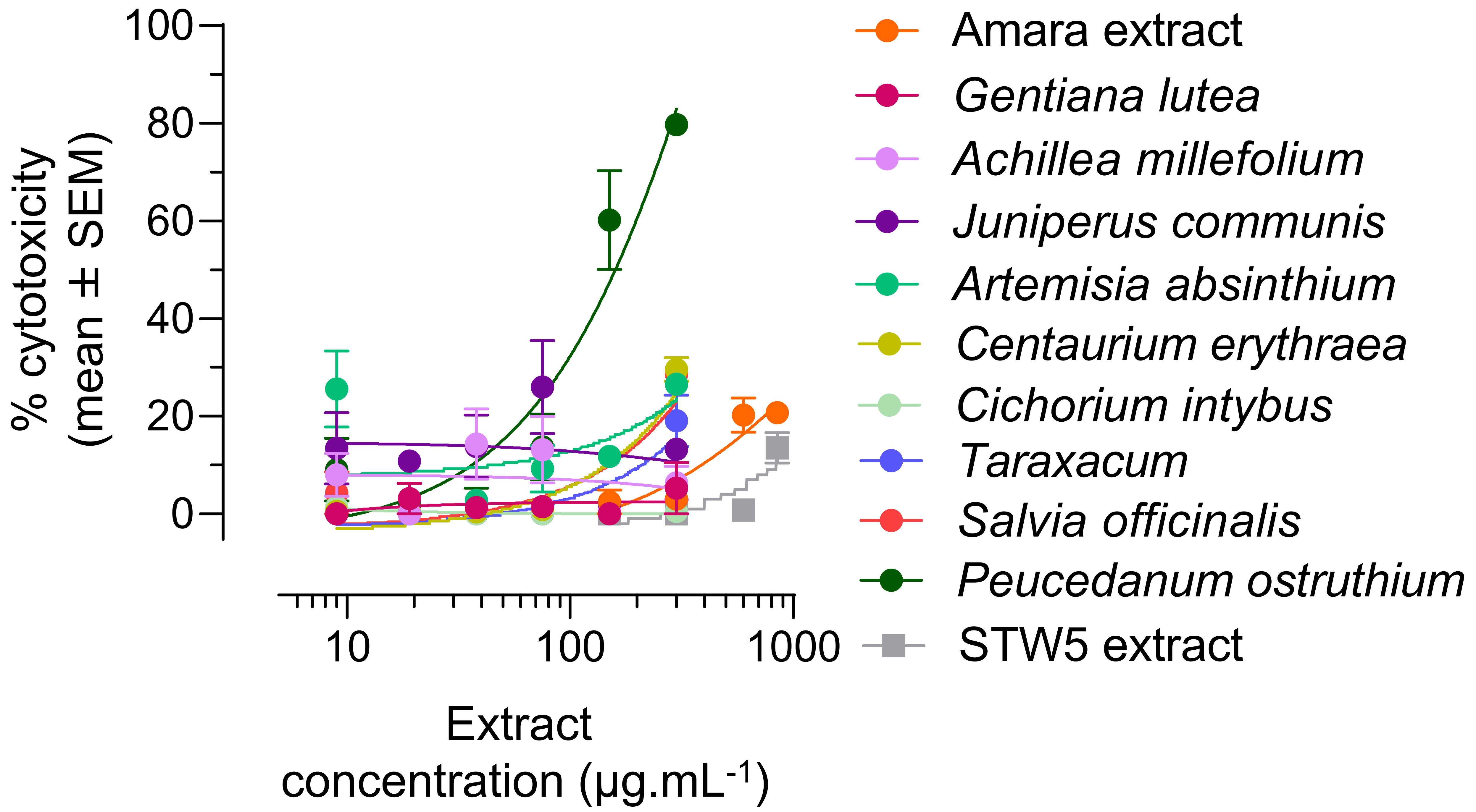

Figure S3. Cytotoxicity of Amara extracts in CHO-K1 cells. CHO-K1-mt aequorin cells were treated with Amara extract or STW5 extract (100, 300, 600 and 850 µg.mL^{-1}) or with Amara individual extracts (9, 19, 38, 75, 150 and 300 µg.mL^{-1}) for 24 hours at 37°C under 5% CO_2. Cytotoxicity was measured using the CellTiter 96® AQueous One Solution Cell Proliferation Assay, and data were expressed as % cytotoxicity relative to the vehicle control. *Peucedanum ostruthium* showed some cytotoxicity at the highest concentrations of 150 and 300 µg.mL^{-1}.

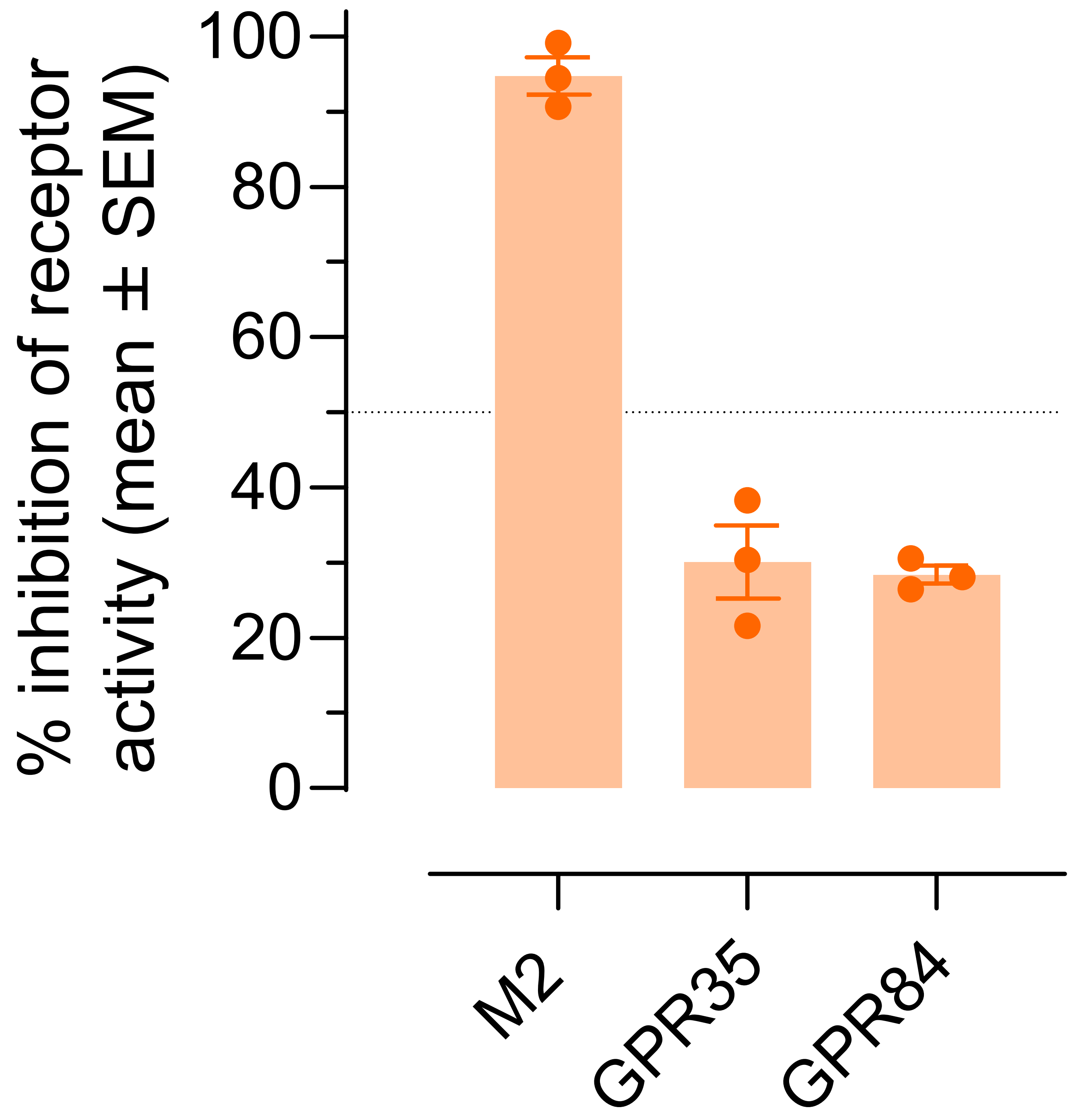

Figure S4. Amara extract does not inhibit the activity of the G protein-coupled receptors GPR35 and GPR84. CHO-K1 cells expressing the recombinant human M2, GPR35 or GPR84 receptor were pre-treated with Amara extract (500 µg.mL^{-1}) or vehicle (0.1% ethanol) and activated by the respective agonist (oxotremorine for M2, zaprinast for GPR35 and capric acid for GPR84) at their EC_{80}. Receptor activity was assessed using the cAMP HTRF assay for Gi-coupled receptors, and results were expressed as the % of inhibition of the reference agonist activity. Amara extract inhibited the M2 but not the GPR35 or GPR84 receptor activity.
