# Supplementary material for: Herbal Amara extract induces gastric fundus relaxation via inhibition of the M2 muscarinic receptor
Source: Neurogastroenterol Motil. 2024 Sep 30;37(1):e14924. doi: 10.1111/nmo.14924 (PMC11650409; doi:10.1111/nmo.14924)
Supplement: Supplementary file 2 — Table S1. Peak intensity of the analytes recorded at the indicated retention times (RT) in electrospray ionization positive mode through UHPLC‐hr‐QToF‐MS/MS analysis (see also Figure S1 A). Table S2. Peak intensity of the analytes recorded at the indicated retention times (RT) in electrospray ionization negative mode through UHPLC‐hr‐QtoF‐MS/MS analysis (see also Figure S1 B). [file NMO-37-e14924-s002.docx]

**Table S1.** Peak intensity of the analytes recorded at the indicated retention times (RT) in electrospray ionization positive mode through UHPLC-hr-QToF-MS/MS analysis (see also Figure S1A).

| **Peak number**† | **Name^§^** | **Type** | **RT (min) / ion (m/z)** | **Peak intensity** |
| --- | --- | --- | --- | --- |
| 1 | Mono caffeoylquinic acid | Esters of caffeic acid with quinic acid | 4.45 / 355.101 | 790 |
|  | Mono caffeoylquinic acid base peak (BP) |  | 4.47 / 163.038 | 29652 |
| 2 | Mono caffeoylquinic acid | Esters of caffeic acid with quinic acid | 6.08 / 355.101 | 40200 |
|  | Mono caffeoylquinic acid BP |  | 6.08 / 163.038 | 289613 |
| 3 | Mono caffeoylquinic acid | Esters of caffeic acid with quinic acid | 6.45 / 355.101 | 4415 |
|  | Mono caffeoylquinic acid BP |  | 6.45 / 163.038 | 68083 |
| 4 | Swertiamarin | Secoiridoid saponin | 6.85 / 397.109 | 13159 |
|  | Swertiamarin BP |  | 6.85 / 195.064 | 179430 |
| 5 | Gentiopicrin | Iridoid glycoside | 7.46 / 357.117 | 139482 |
|  | Gentiopicrin BP |  | 7.46 / 195.064 | 732780 |
| 6 | Tuberonic acid glucoside | Acid glucoside | 7.62 / 411.161 | 65701 |
| 7 | Sweroside | Secoiridoid glycoside | 7.71 / 381.114 | 4243 |
| 8 | Rosmarinic acid | Phenolic compound | 7.73 / 361.09 | 5345 |
| 9 | Apigenin glucopyranoside | Flavonoid glycoside | 7.9 / 595.165 | 6401 |
| 10 | Tuberonic acid | Oxo monocarboxylic acid | 8.38 / 209.116 | 15273 |
| 12 | Quercetin glucuronide | Flavonoid | 8.78 / 479.08 | 12181 |
| 13 | Kaempferol rutinoside | Flavonoid | 9.86 / 595.163 | 5123 |
| 14 | Tetrahydroxyflavone e.g. Luteolin BP | Flavonoid | 9.96 / 287.054 | 21091 |
|  | Luteolin glucuronide |  | 9.96 / 463.086 | 99161 |
| 15 | Dicaffeoylquinic acid | Quinic acid | 10.36 / 499.122 | 1771 |
|  | Dicaffeoylquinic acid BP |  | 10.36 / 163.038 | 13581 |
| 16 | Dicaffeoylquinic acid (M-H2O)+ | Quinic acid | 10.51 / 499.122 | 14288 |
|  | Dicaffeoylquinic acid BP |  | 10.51 / 163.038 | 61312 |
| 24 | Nepetin glucoside | Flavonoid glucoside | 10.57 / 479.117 | 6589 |
| 25 | Dicaffeoylquinic acid BP | Quinic acid | 10.69 / 163.038 | 59746 |
| 26 | Dicaffeoylquinic acid | Quinic acid | 10.76 / 539.114 | 3273 |
|  | Dicaffeoylquinic acid (M-H2O)+ |  | 10.78 / 499.122 | 20517 |
|  | Dicaffeoylquinic acid BP |  | 10.78 / 163.038 | 54763 |
| 27 | Apigenin/Genistein glucuronide | Flavonoids | 11.17 / 447.091 | 23789 |
|  | Apigenin glucoside | Flavonoid glucoside | 11.17 / 433.112 | 42102 |
| 31 | Cynarin (M-H2O)+ | Ester of a hydroxycinnamic acid | 11.41 / 499.122 | 26624 |
| 32 | Hispidulin 7-glucuronide | Flavonoid | 11.67 / 477.101 | 18136 |
| Standard | Amarogentin | Biphenolic acid ester of gentiopicrin | 12.76 / 587.175 | 13014 |
| 33 | Methyl catechin fragment | Flavonoid | 13.8 / 203.032 | 16548 |
|  | Methyl catechin |  | 13.8 / 305.101 | 341072 |
| 34 | Apigenin | Flavonoid | 15.31 / 271.058 | 19388 |
| 35 | iso-rosmanol | Diterpene lactone | 19.26 / 347.184 | 55883 |
|  | iso-rosmanol BP |  | 19.26 / 301.179 | 129308 |
| 36 | Deoxylactucin | Sesquiterpene lactone | 22.33 / 261.11 | 8554 |
| 37 | C_16_H_14_O_4_ e.g. Imperatorin | Furocoumarin | 23.18 / 271.095 | 100891 |
| 38 | Rosmadial fragment | Phenolic diterpene lactone | 24.26 / 233.043 | 22017 |
|  | Rosmadial (C_20_H_24_O_5_) |  | 24.29 / 345.169 | 210885 |
| 39 | C_16_H_14_O_4_ e.g. Isoimperatorin | Psoralen | 25.09 / 293.077 | 11503 |
| 40 | Dimethyl rosmanol | Phenolic diterpene lactone | 27.27 / 375.216 | 238454 |
| 41 | C_19_H_22_O_3_ e.g. Ostruthin | Terpene lactone | 28.83 / 299.163 | 110839 |

† Eleven of the 41 detected secondary metabolites (number 11, 17-23, 28-30) could not be annotated. ^§^ Fields marked in blue indicate secondary metabolites with a known function in gastrointestinal protection, regulation or motility.

**Table S2.** Peak intensity of the analytes recorded at the indicated retention times (RT) in electrospray ionization negative mode through UHPLC-hr-QToF-MS/MS analysis (see also Figure S1B).

| **Peak number** | **Name^§^** | **Type** | **RT (min) / ion (m/z)** | **Peak intensity** |
| --- | --- | --- | --- | --- |
| 1 | Mono caffeoylquinic acid | Esters of caffeic acid with quinic acid | 5.93 / 353.086 | 78828 |
|  | Mono Caffeoylquinic acid BP |  | 5.93 / 191.056 | 225954 |
| 2 | Mono caffeoylquinic acid | Esters of caffeic acid with quinic acid | 6.31 / 353.086 | 23647 |
| 3 | Swertiamarin | Secoiridoid saponin | 6.82 / 419.117 | 18306 |
| 4 | Gentiopicrin | Iridoid glycoside | 7.42 / 401.107 | 121716 |
|  | Gentiopicrin BP |  | 7.42 / 179.056 | 150603 |
| 5 | Tuberonic acid glucoside | Acid glucoside | 7.6 / 387.164 | 82258 |
| 6 | Cynarin | Ester of a hydroxycinnamic acid | 7.71 / 515.117 | 4485 |
| 7 | Kaempferol rutinoside | Flavonoid | 9.81 / 593.148 | 4053 |
| 8 | Luteolin glucuronide | Flavonoid | 9.85 / 461.07 | 52502 |
| 9 | Dicaffeoylquinic acid | Quinic acid | 10.38 / 515.117 | 21800 |
| 10 | Nepetin glucoside | Flavonoid glucoside | 10.49 / 477.101 | 6053 |
| 11 | Dicaffeoylquinic acid | Quinic acid | 10.55 / 515.117 | 11316 |
| 12 | Dicaffeoylquinic acid | Quinic acid | 10.64 / 515.117 | 10347 |
|  | Dicaffeoylquinic acid BP |  | 10.64 / 353.086 | 23398 |
| 13 | Apigenin glucuronide / Genistein glucuronide | Flavonoids | 11.03 / 445.075 | 9203 |
|  | Genistein glucuronide / Apigenin glucuronide BP |  | 11.06 / 431.096 | 43255 |
| 14 | Dicaffeoylquinic acid fragment | Quinic acid | 11.26 / 353.086 | 9218 |
|  | Dicaffeoylquinic acid |  | 11.26 / 515.117 | 56315 |
| 15 | Hispidulin glucuronide | Flavonoid | 11.53 / 475.085 | 6572 |
| Standard | Amarogentin | Biphenolic acid ester of gentiopicrin | 12.64 / 585.159 | 21852 |
| 16 | Tetrahydroxyflavone e.g. Luteolin, Kaempferol | Flavonoid | 13.37 / 285.039 | 66728 |
| 17 | Caffeic acid ethyl ester | Ester of a hydroxycinnamic acid | 13.85 / 207.066 | 114368 |
| 18 | Apigenin | Flavonoid | 15.03 / 269.044 | 74004 |
| 19 | Trihydroxy methoxyflavone e.g. Hispidulin | Monomethoxyflavone | 15.52 / 299.055 | 76733 |
| 20 | C_20_H_24_O_5_ e.g. Rosmadial | Phenolic diterpene lactone | 23.92 / 343.154 | 942572 |
| 21 | Dimethyl rosmanol | Phenolic diterpene lactone | 26.92 / 373.201 | 316280 |
| 22 | C_19_H_22_O_3_ e.g. Ostruthin | Terpene lactone | 28.43 / 297.149 | 532905 |
| 23 | C_21_H_30_O_4_ e.g. Pinusolide | Diterpene lactone | 30.11 / 345.206 | 553642 |

^§^ Fields marked in blue indicate secondary metabolites with a known function in gastrointestinal protection, regulation or motility (see text).
