# Supplementary material for: Herbal Amara extract induces gastric fundus relaxation via inhibition of the M2 muscarinic receptor
Source: Neurogastroenterol Motil. 2024 Sep 30;37(1):e14924. doi: 10.1111/nmo.14924 (PMC11650409; doi:10.1111/nmo.14924)
Supplement: Supplementary file 3 — Data S1: Supporting Information. [file NMO-37-e14924-s001.pdf]

SUPPORTIVE INFORMATION - DETAILED MATERIALS AND METHODS

1. | Herbal extracts

Amara Drops (originally described in the monograph of the Commission C entitled "Cichorium/Taraxacum comp." published in the German Federal Gazette Nr. 99a in June 1986, and amended in the Federal Gazette Nr. 85 in May 1991)[1] is a 33% (vol) ethanol solution containing the following amounts of tinctures (in g) for 10 g (10.4 mL) of product: 0.15 g ethanolic extract from *Artemisia absinthium*, Herba rec. (1:2.3); 0.075 g ethanolic extract from *Centaurium erythraea*, Herba rec. (1:2.3); 0.6 g ethanolic extract from *Cichorium intybus*, Planta tota rec. (1:2.3); 0.36 g ethanolic decoction from *Gentiana lutea*, Rhizoma and Radix Ø (Ph. Eur. 10/2544, V. 1.2.10); 0.05 g ethanolic infusion from *Juniperus communis*, Summitates Ø (Ph. Eur. 10/2545, V. 1.2.13); 2.0 g ethanolic infusion from *Achillea millefolium* Ø (Ph. Eur. 10/2545, V. 1.2.13); 0.15 g ethanolic decoction from *Peucedanum ostruthium*, Rhizoma rec. (1:2.15) (Ph. Eur. 10/2544, V. 1.2.10); 1.0 g ethanolic infusion from Salvia officinalis Ø (Ph. Eur. 10/2545, V. 1.2.13); 0.32 g *Taraxacum*, Planta tota rec. Ø (Ph. Eur. 10/2535, V. 1.1.3).[2] A recommended dosage of Amara Drops (15 drops or 348 µl) contains a total of 3.97 mg dry herbal extract, including the following amounts of the respective dry herbal components: 152 µg *Artemisia absinthium*, 66 µg *Centaurium erythraea*, 344 µg *Cichorium intybus*, 571 µg *Gentiana lutea*, 37 µg *Juniperus communis*, 1416 µg *Achillea millefolium*, 126 µg *Peucedanum ostruthium*, 910 µg Salvia officinalis, and 345 µg *Taraxacum*.

The nine individual extract tinctures were prepared as follows: (1) *Artemisia absinthium*, Herba rec., ethanolic extract 1:2.3 EtOH 30% (m/m); (2) *Centaurium erythraea*, Herba rec., ethanolic extract 1:2.3 EtOH 30% (m/m); (3) *Cichorium intybus*, Planta tota rec., ethanolic extract 1:2.3 EtOH 30% (m/m); (4) *Gentiana lutea*, Rhizoma and Radix, ethanol.Decoctum Ø (Ph. Eur. 10/2544, V. 1.2.10); (5) *Juniperus communis*, Summitates, ethanol.Infusum Ø 1:10 (Ph. Eur. 10/2545, V. 1.2.13) EtOH 30% (m/m); (6) *Achillea millefolium*, ethanol.Infusum Ø 1:10 (Ph. Eur. 10/2545, V. 1.2.13) EtOH 30% (m/m); (7) *Peucedanum ostruthium*, Rhizoma rec., ethanol.Decoctum (Ph. Eur. 10/2544, V. 1.2.10) 1:2.15 EtOH 43% (m/m); (8) *Salvia officinalis*,

ethanol.Infusum Ø 1:10 (Ph. Eur. 10/2545, V. 1.2.13) EtOH 62% (m/m); (9) *Taraxacum*, Planta tota rec. Ø (Ph. Eur. 10/2535, V. 1.1.3).

All nine respective plants were organically cultivated in Germany on fields dedicated to medicinal plant cultivation.

For all experiments, dry extracts of Amara were prepared from one same batch of Amara Drops (Weleda AG, Schwäbisch Gmünd, Germany) and stored at room temperature until use. Dry extracts of individual herbal extracts entering in the composition of Amara extract were prepared from different batches of the same tinctures used to prepare Amara Drops.

Before experimental use, Amara and individual extract stock solutions (thereafter referred to as "Amara extract" and "Amara individual extracts", respectively) were prepared as follows:

For the HPLC experiment, Amara dry extract was dissolved in 50% aqueous ethanol (w/v) at 1 $mg.mL^{-1}$, vortexed for two minutes, ultrasonicated for five minutes, and ultracentrifuged at 13,500 x g for 10 minutes. The supernatant was carefully harvested and spiked with 1.55 $mg.L^{-1}$ amarogentin (80178; PhytoLab GmbH, Vestenbergsgreuth, Germany) as internal standard for data normalization prior to instrumental analysis.

For the organ bath experiment, Amara dry extract was dissolved in physiological saline solution (PSS; 119 mM NaCl, 4.7 mM KCl, 1.2 mM $MgSO_4$, 24.9 mM $NaHCO_3$, 1.2 mM KH_2PO_4, 2.5 mM $CaCl_2$ and 11.1 mM glucose) at 30 $mg.mL^{-1}$, vortexed for 5 – 10 minutes, sonicated for 30 minutes with occasional vortexing, and centrifuged at 3000 x g for 10 minutes. The supernatant was carefully harvested and used within 24 hours for organ bath experiments. PSS served as vehicle control.

For M2 and M3 receptor inhibition assays in CHO-K1 cells, Amara dry extract was dissolved in 50% aqueous ethanol (w/v) at 120 and 60 $mg.mL^{-1}$, respectively, vortexed for 5 – 10 minutes, sonicated for 30 minutes with occasional vortexing, and centrifuged at 3000 x g for 10 minutes. The supernatant was carefully harvested and used within 24 hours in cell culture experiments at the indicated concentrations (in 0.1% ethanol final dilution in culture medium without antibiotics). Culture medium was composed of Ham's F12 (21765, Gibco/ThermoFisher Scientific, Waltham, MA, USA), 10% fetal bovine serum (10270-106, Gibco), 100 IU/ml

penicillin, 100 µg.mL^{-1} streptomycin (DE17-602E, Lonza, Basel, Switzerland), and 400 µg.mL^{-1} Geneticin (10131-027, Gibco). Culture medium containing 0.1% ethanol served as vehicle control.

For the cytotoxicity assay in CHO-K1 cells, Amara dry extract was dissolved in 50% aqueous ethanol (w/v) at 150 mg.mL^{-1}, vortexed for 5 – 10 minutes, sonicated for 30 minutes with occasional vortexing, and centrifuged at 3000 x g for 10 minutes. The supernatant was carefully harvested and used within 24 hours in the cytotoxicity assay, diluted in culture medium without antibiotics. Culture medium containing 0.1% ethanol served as vehicle control.

For the radioligand M2 binding assay, Amara dry extract was dissolved in in 50% aqueous ethanol (w/v) at 200 mg.mL^{-1}, vortexed for 5 – 10 minutes, sonicated for 30 minutes with occasional vortexing, and centrifuged at 3000 x g for 10 minutes. The supernatant was carefully harvested and used within 24 hours for the binding assay at the indicated concentrations in Binding Buffer (50 mM Tris-HCl pH 7.4, 1 mM EDTA, 10 µg.mL^{-1} saponine) containing 0.1% ethanol final. Binding Buffer containing 0.1% ethanol served as vehicle control.

The herbal extract STW5 (Iberogast Classic, Bayer Vital GmbH, **Leverkusen**, Germany) was used as a control in some experiments.[3–7] A STW5 lyophilisate was prepared and stored at room temperature until use. Before experimental use, STW5 extract stock solutions (thereafter referred to as “STW5 extract”) were prepared in the same conditions as those described above for Amara extracts.

2. | Composition analysis of Amara extract

The composition of Amara extract was evaluated by ultra-high-performance liquid chromatography with high-resolution quadrupole time-of-flight tandem mass spectrometry (UHPLC-hr-QToF-MS/MS) using a Dionex UltiMate 3000 HPLC system (ThermoFisher Scientific, Waltham, MA, USA) coupled with a maXis Impact Ultra High Resolution ToF-MS mass spectrometer (Bruker Daltonics, Bremen, Germany). A Zorbax Rapid Resolution High Definition (RRHD) C18 column (100 mm length x 2.1 inner diameter, 1.8 µm particle size; Agilent, Santa Clara, CA, USA) was used for chromatographic separation. The mobile phase consisted in acetonitrile (B) and 0.1% formic acid in water (A). The flow rate was 0.4 mL.min^{-1}, injection volume was 1 µl, and the column oven was set at 45°C. The following gradient was used: 0.0 min 3% B, 28.0 min 65% B, 30.0

min 100% B, 32.0 min 100% B, 32.5 min 3% B, 35.0 min 3% B.

The eluate from liquid chromatography was directly introduced into the mass spectrometer with mass scanning from 50 – 1000 *m/z* and spectra rate 4 Hz, using electrospray ionization (ESI) in positive and negative full scan and MS/MS modes. The mass accuracy before each run was verified by comparison with sodium formate adducts. The mass accuracies were rounded to 1 mDa, and the corresponding retention times to 0.05 min. The UV spectra were recorded at 280 nm.

Interpretation of the mass signals was conducted using the Compass Data Analysis 4.2 and MetaboScape 5.0 softwares (Bruker, Billerica, MA, USA). Peak annotation was performed using the National Institute of Standards and Technology (NIST) Mass Spectral Library (U.S. Department of Commerce, Gaithersburg, MD, USA). Moreover, manual annotation of the chromatographic and mass spectrometric data was achieved through available literature citations.[8–13] Each sample was measured as technical duplicates.

3. | Guinea-pig fundus motility assays

Guinea-pig fundus motility experiments were conducted by REPROCELL Europe (Glasgow, UK) under the license number XC2FD842E granted by the University of Glasgow Ethics Committee on 30 April 1987, amended on 26 September 2023, and approved by the User Research Ethics Board of the UK Home Office. Strips of circular muscle were dissected from the fundus of male adult Dunkin Hartley guinea pigs. Only tissues passing viability checks were used. Data are mean ± standard error of the mean (SEM) of at least three independent experiments, each performed using up to two animals. Depending on tissue availability per animal, several strips (or replicates) were used in each experiment, as indicated in the respective figure legends.

Fundus circular muscle strips of approximately 15 mm long and 2 – 3 mm wide were dissected from surrounding tissue and mucosa was removed. Fundus circular muscle strips were mounted in individual 25 mL organ baths containing PSS and maintained at 37°C under 95% O_2 / 5% CO_2 throughout the experiment. Changes in force production were recorded using transducers (TRI202PAD, Panlab Harvard Apparatus, Barcelona, Spain). After mounting in organ baths, the fundus muscle strips were equilibrated in PSS for 30

minutes before they were set to a stable tension of 1.0 g ± 0.2 g. Tissues were then allowed to equilibrate over 45 minutes with washes every 15 minutes, until stabilization of baseline tension.

For the smooth muscle relaxation experiment, the muscle strips were first exposed to 1 µM carbachol (Sigma-Aldrich/Merck, Gillingham, UK) for approximately 5 minutes or until the plateau of response was achieved, to control for muscle responsiveness. Muscles strips were then washed three times with PSS to allow the response to reach baseline levels. Upon stabilization of the baseline tone, the fundus muscle strips were exposed to either PSS (vehicle), isoprenaline (100 pM – 10 µM; Sigma-Aldrich/Merck) as smooth muscle relaxant control,[14–16] or Amara extract (3.5 – 1000 µg.mL^{-1}). Vehicle bath received the same volume of PSS as the Amara extract baths. Incubations were conducted for a minimum of 20 minutes or until plateau of response. Papaverine (100 µM; Sigma-Aldrich/Merck) was then added to all organ baths to induce complete smooth muscle relaxation.[17,18] Papaverine-induced muscle relaxation post-assay also served as a viability test to control for the absence of tissue toxicity. The magnitude of response was measured by determining the lowest or highest point of response at each concentration of the curve, as appropriate. Changes in baseline tone at each concentration were compared to the baseline tone before the curve, and data were expressed as the percentage of change of the baseline tone response.

For the cumulative concentration response curve to Amara extract following carbachol-induced fundus muscle contraction, muscle strips were first exposed to 10 µM carbachol until the plateau of constriction response was achieved. Organ baths were then washed three times with PSS to allow the response to reach baseline levels. Upon stabilization of the baseline tone, organ baths were pre-treated with the M3 receptor antagonist J-104129 (300 nM; Bio-Techne, Abingdon, UK)[19,20] or vehicle (0.03% DMSO) for 30 minutes. J-104129 has a 120-fold selectivity for M3 receptors (K_i = 4.2 nM) over M2 receptors (K_i = 490 nM),[19] and the applied concentration of 300 nM for J-104129 is expected to strongly inhibit M3 without interfering with M2 receptor activity. Smooth muscle constriction was then induced by 10 µM carbachol until plateau of response before exposure to vehicle (PSS) or Amara extract (94.4 – 1000 µg.mL^{-1}) for 10 – 15 minutes or until plateau of response. Papaverine (100 µM) was then added to all organ baths to induce complete smooth muscle relaxation, as a control for tissue viability post-assay. The magnitude of response was measured at each

concentration of the curve and data were expressed as the percentage of change of carbachol-induced constriction.

To control the potency of the M3 receptor antagonist J-104129 to partially inhibit carbachol-induced fundus muscle contraction, muscle strips were first exposed to 10 µM carbachol until the plateau of constriction response was achieved. Organ baths were then washed three times with PSS to allow the response to reach baseline levels. Upon stabilization of the baseline tone, the fundus muscle strips were pre-treated with either 300 nM J-104129 or 0.03% DMSO (vehicle) for 30 minutes, before a second exposure to 10 µM carbachol to induce constriction, until the plateau of response. Muscle contraction (tension) was measured and expressed as gram (g) tension.

4. | M2 muscarinic receptor inhibition assay in CHO-K1 recombinant cell line

The inhibitory effect of Amara extract on M2 muscarinic receptor activity was evaluated in CHO-K1 cells expressing the recombinant human M2 receptor (accession number NP_000730.1) using the cAMP HTRF assay for Gi-coupled receptors (FAST-0261C; EuroscreenFast, Charleroi, Belgium), as described by the manufacturer. Briefly, CHO-K1 cells expressing recombinant human M2 receptor grown in medium without antibiotic were detached by gentle flushing with phosphate buffer saline (PBS) containing 5 mM EDTA, recovered by centrifugation and resuspended in KRH assay buffer (5 mM KCl, 1.25 mM $MgSO_4$, 124 mM NaCl, 25 mM HEPES, 13.3 mM Glucose, 1.25 mM KH_2PO_4, 1.45 mM $CaCl_2$, 0.5 $g.L^{-1}$ bovine serum albumin [BSA], supplemented with 1mM of the non-selective phosphodiesterase inhibitor IBMX). Cells (triplicate conditions) were pre-incubated for 10 minutes with increasing concentrations (9 – 600 $\mu g.mL^{-1}$ in 0.1% ethanol) of Amara extract or Amara individual extracts, or vehicle (0.1% ethanol), before activation of the M2 receptor by forskolin and the M2 agonist oxotremorine (at its historical EC_{80} concentration: 71 nM). After incubation at room temperature for 30 minutes, cells were lysed in buffer containing cAMP-d2 and anti-cAMP cryptate detection reagents for 1 hour at room temperature, and fluorescence ratios were measured with the Homogeneous Time Resolved Fluorescence (HTRF) kit according to the manufacturer's specification. Dose-response data were expressed as a percentage of inhibition of oxotremorine-induced M2 activation.

Control cAMP HTRF assays for the Gi-coupled receptors GPR35 (FAST-0915C) and GPR84 (FAST-0935C) were conducted as described above, following the manufacturer's recommendations. Cells were pre-incubated for 10 minutes with Amara extract (500 µg.mL^{-1}) or vehicle (0.1% ethanol) and activated with the respective agonist (3370 µM zaprinast for GPR35, 5350 µM capric acid for GPR84) for 30 minutes at room temperature. Cells were lysed and fluorescence ratios measured as described above. Data were expressed as a percentage of inhibition of agonist-induced receptor activation.

5. | M3 muscarinic receptor inhibition assay in CHO-K1 recombinant cell line

The inhibitory effect of Amara extract on M3 muscarinic receptor activity was evaluated in CHO-K1-mt aequorin cells expressing the recombinant human M3 receptor (accession number NP_000731.1) using the IPOne HTRF assay (ES-212A; EuroscreenFast), as described by the manufacturer. Briefly, CHO-K1 cells expressing recombinant human M3 receptor grown in medium without antibiotic were detached by gentle flushing with PBS – 5 mM EDTA, recovered by centrifugation and resuspended in medium without antibiotics. Cells were distributed in a 96-well plate (20,000 cells per well in triplicate conditions) and incubated overnight at 37°C under 5% CO_2. Amara or STW5 extracts (38 – 600 µg.mL^{-1} in 0.1% ethanol), or vehicle (0.1% ethanol) were added to the cells and incubated for 15 minutes at 37°C under 5% CO_2 before activation of the M3 receptor by the M3 agonist acetylcholine (at its historical EC_{80} concentration: 500 nM). After incubation at 37°C under 5% CO_2 for 1 hour, cells were lysed in buffer containing IP1-d2 and anti-IP1 cryptate detection reagents for 1 hour at room temperature, and fluorescence ratios were measured with the HTRF kit according to the manufacturer's specification. Dose-response data were expressed as a percentage of inhibition of acetylcholine-induced M3 activation.

6. | Cytotoxicity control assay

Cytotoxicity assays were conducted by EuroscreenFast in the CHO-K1-mt aequorin cell line expressing the human recombinant M3 receptor (see **2.5**). Briefly, CHO-K1-mt aequorin cells grown in medium without antibiotic were detached by gentle flushing with PBS – 5 mM EDTA, centrifuged, resuspended in medium without antibiotics, seeded as three replicates in a 96-well culture plate, and incubated for 24 hours at 37°C

under 5% CO_2. Amara extract (100 – 850 µg.mL^{-1}), STW5 extract (100 – 850 µg.mL^{-1}), Amara individual extracts (9 – 300 µg.mL^{-1}), or vehicle (0.1% ethanol in medium) were added to the cells and incubated for 1.5 hours at 37°C under 5% CO_2. Cytotoxicity was evaluated using the CellTiter 96® AQueous One Solution Cell Proliferation Assay (Promega, Madison, WI, USA) according to the manufacturer's instructions. Absorbance was measured at 490 nm using an ELISA plate reader (SpectraMax Plus 384, Molecular Devices, San Jose, USA), and data were expressed as % cytotoxicity relative to the vehicle control.

7. | Radioligand M2 binding competition assay

Binding of Amara extract to the M2 muscarinic receptor was tested by radioligand binding competition assay (FAST-0261B; EuroscreenFast), as recommended by the manufacturer, using CHO-K1 cell membrane extracts. Membrane extracts were prepared from mid-log phase grown cells harvested in buffer A (15 mM Tris-HCl pH 7.5, 2 mM $MgCl_2$, 0.3 mM EDTA, 1 mM EGTA) and homogenised in a glass-glass homogenizer. The crude membrane fraction was collected by two consecutive centrifugations at 35,000 x g at 4°C for 30 minutes separated by a washing step in buffer A. The final membrane fraction was resuspended in buffer B (75 mM Tris-HCl pH 7.5, 12.5 mM $MgCl_2$, 0.3 mM EDTA, 1 mM EGTA, 250 mM sucrose) and flash-frozen in liquid nitrogen. Protein content was determined by the bicinchoninic acid (BCA) method (UP40840A, Interchim, Montluçon, France). The competition binding assay was performed in duplicate in a 96-well plate (MASTERBLOCK 786201, Greiner Bio-One, Frickenhausen, Germany) containing Binding Buffer (50 mM Tris-HCl pH 7.4, 1 mM EDTA, 10 µg.mL^{-1} saponine), membrane extracts (4 µg protein/well), radiotracer (0.5 nM [^{3}H] N-methylscopolamine), and increasing concentrations of Amara extract (19 – 600 µg.mL^{-1}). Non-specific binding was determined by co-incubation with a 200-fold excess of cold competitor ligand (N-methylscopolamine). Samples were incubated in a final volume of 0.1 mL at room temperature for 1 hour and then filtered over filter plates. Filters were washed six times with 0.5 mL of ice-cold Washing Buffer (50 mM Tris-HCl pH 7.4), and 50 µl of MicroScint-20 scintillation cocktail (Perkin Elmer, Waltham, MA, USA) were added in each well. The plates were incubated 15 minutes on an orbital shaker and radiolabelled signal was measured for 1 min/well using a TopCount microplate scintillation counter (Perkin Elmer). Data were

expressed as the percentage of residual binding of the radiotracer or “control activator” (N-methylscopolamine) to the M2 receptor.

8. | Statistical analysis

All analysed data were displayed graphically using GraphPad Prism version 9.2.0. (GraphPad Software Inc., San Diego, CA, USA). Statistical analysis of organ bath data was performed using the two-way ANOVA with Dunnett’s post-hoc test in GraphPad Prism version 9.2.0., to compare the effect of Amara to that of the vehicle control, taking into account variable number of replicates per condition. A p-value ≤ 0.05 was considered statistically significant. IC_{50} values of dose-response experiments (receptor activity and binding assays) were determined with the XLfit software version 5.5.0 (IDBS, Woking, UK) using nonlinear regression applied to a sigmoidal dose-response model (XL Fit fit Model 203). Cytotoxicity ≤ 20% relative to the vehicle control was not considered as significant.

REFERENCES

1. Cichorium / Taraxacum comp. Commission C Monograph. In: *Anthroposophical Medicine*. Society of Anthroposophical Doctors in Germany on behalf of the Medical Section at the Goetheanum Dornach/Switzerland; 1999:370-371.

2. Weleda AG. AMARA-TROPFEN. AMARA-TROPFEN Leaflet. Published 2013. Accessed September 21, 2023. https://www.weleda.de/restservices-deu-de/ppis/downloadPdf/322400_Leaflet

3. Hohenester B, Rühl A, Kelber O, Schemann M. The herbal preparation STW5 (lberogast) has potent and region-specific effects on gastric motility. *Neurogastroenterol Motil*. 2004;16(6):765-773. doi:10.1111/j.1365-2982.2004.00548.x

4. Melzer J, Iten F, Reichling J, Saller R. Iberis amara L. and Iberogast®– a systematic review concerning dyspepsia. *Focus on Alternative and Complementary Therapies*. 2003;8(4):518-518. doi:10.1111/j.2042-7166.2003.tb04037.x

5. Rösch W, Liebregts T, Gundermann KJ, Vinson B, Holtmann G. Phytotherapy for functional dyspepsia: a review of the clinical evidence for the herbal preparation STW 5. *Phytomedicine*. 2006;13 Suppl 5:114-121. doi:10.1016/j.phymed.2006.03.022

6. Schemann M, Michel K, Zeller F, Hohenester B, Rühl A. Region-specific effects of STW 5 (Iberogast) and its components in gastric fundus, corpus and antrum. *Phytomedicine*. 2006;13 Suppl 5:90-99. doi:10.1016/j.phymed.2006.03.020

7. Wegener T, Wagner H. The active components and the pharmacological multi-target principle of STW 5 (Iberogast). *Phytomedicine*. 2006;13 Suppl 5:20-35. doi:10.1016/j.phymed.2006.07.001

8. Dall'Acqua S, Bolego C, Cignarella A, Gaion RM, Innocenti G. Vasoprotective activity of standardized Achillea millefolium extract. *Phytomedicine*. 2011;18(12):1031-1036. doi:10.1016/j.phymed.2011.05.005

9. Yilmaz MA, Ertas A, Yener I, et al. A comprehensive LC-MS/MS method validation for the quantitative investigation of 37 fingerprint phytochemicals in Achillea species: A detailed examination of A. coarctata and A. monocephala. *J Pharm Biomed Anal*. 2018;154:413-424. doi:10.1016/j.jpba.2018.02.059

10. Rahmani Samani M, D'Urso G, Montoro P, Ghasemi Pirbalouti A, Piacente S. Effects of bio-fertilizers on the production of specialized metabolites in Salvia officinalis L. leaves: An analytical approach based on LC-ESI/LTQ-Orbitrap/MS and multivariate data analysis. *J Pharm Biomed Anal*. 2021;197:113951. doi:10.1016/j.jpba.2021.113951

11. Bergantin C, Maietti A, Cavazzini A, et al. Bioaccessibility and HPLC-MS/MS chemical characterization of phenolic antioxidants in Red Chicory (Cichorium intybus). *Journal of Functional Foods*. 2017;33:94-102. doi:10.1016/j.jff.2017.02.037

12. Aberham A, Pieri V, Croom EM, Ellmerer E, Stuppner H. Analysis of iridoids, secoiridoids and xanthones in Centaurium erythraea, Frasera caroliniensis and Gentiana lutea using LC-MS and RP-HPLC. *J Pharm Biomed Anal*. 2011;54(3):517-525. doi:10.1016/j.jpba.2010.09.030

13. Jedrejek D, Lis B, Rolnik A, Stochmal A, Olas B. Comparative phytochemical, cytotoxicity, antioxidant and haemostatic studies of Taraxacum officinale root preparations. *Food Chem Toxicol*. 2019;126:233-247. doi:10.1016/j.fct.2019.02.017

14. Kim KS, Shim WS, dela Peña IC, et al. Smooth muscle relaxation activity of an aqueous extract of dried immature fruit of Poncirus trifoliata (PF-W) on an isolated strip of rat ileum. *Nat Prod Commun*. 2013;8(8):1143-1148.

15. Ito Y, Takagi K, Tomita T. Relaxant actions of isoprenaline on guinea-pig isolated tracheal smooth muscle. *British Journal of Pharmacology*. 1995;116(6):2738-2742. doi:10.1111/j.1476-5381.1995.tb17235.x

16. Baker DM, Watson SP, Santer RM. Evidence for a decrease in sympathetic control of intestinal function in the aged rat. *Neurobiol Aging*. 1991;12(4):363-365. doi:10.1016/0197-4580(91)90023-d

17. Heinle H, Hagelauer D, Pascht U, Kelber O, Weiser D. Intestinal spasmolytic effects of STW 5 (Iberogast) and its components. *Phytomedicine*. 2006;13 Suppl 5:75-79. doi:10.1016/j.phymed.2006.03.013

18. Izzo AA, Mascolo N, Costa M, Capasso F. Effect of papaverine on synaptic transmission in the guinea-pig ileum. *Br J Pharmacol*. 1997;121(4):768-772. doi:10.1038/sj.bjp.0701142

19. Mitsuya M, Mase T, Tsuchiya Y, et al. J-104129, a novel muscarinic M3 receptor antagonist with high selectivity for M3 over M2 receptors. *Bioorganic & Medicinal Chemistry*. 1999;7(11):2555-2567. doi:10.1016/S0968-0896(99)00177-7

20. Mitsuya M, Ogino Y, Kawakami K, et al. Discovery of a muscarinic M3 receptor antagonist with high selectivity for M3 over M2 receptors among 2-[(1S,3S)-3-sulfonylaminocyclopentyl]phenylacetamide derivatives. *Bioorg Med Chem*. 2000;8(4):825-832. doi:10.1016/s0968-0896(00)00008-0
